# Gene Expression Responses to FUS, EWS, and TAF15 Reduction and Stress Granule Sequestration Analyses Identifies FET-Protein Non-Redundant Functions

**DOI:** 10.1371/journal.pone.0046251

**Published:** 2012-09-25

**Authors:** Jenny Blechingberg, Yonglun Luo, Lars Bolund, Christian Kroun Damgaard, Anders Lade Nielsen

**Affiliations:** 1 Department of Biomedicine, Aarhus University, Aarhus, Denmark; 2 Department of Molecular Biology and Genetics, Aarhus University, Aarhus, Denmark; National Institute of Health, United States of America

## Abstract

The FET family of proteins is composed of FUS/TLS, EWS/EWSR1, and TAF15 and possesses RNA- and DNA-binding capacities. The FET-proteins are involved in transcriptional regulation and RNA processing, and FET-gene deregulation is associated with development of cancer and protein granule formations in amyotrophic lateral sclerosis, frontotemporal lobar degeneration, and trinucleotide repeat expansion diseases. We here describe a comparative characterization of FET-protein localization and gene regulatory functions. We show that FUS and TAF15 locate to cellular stress granules to a larger extend than EWS. FET-proteins have no major importance for stress granule formation and cellular stress responses, indicating that FET-protein stress granule association most likely is a downstream response to cellular stress. Gene expression analyses showed that the cellular response towards FUS and TAF15 reduction is relatively similar whereas EWS reduction resulted in a more unique response. The presented data support that FUS and TAF15 are more functionally related to each other, and that the FET-proteins have distinct functions in cellular signaling pathways which could have implications for the neurological disease pathogenesis.

## Introduction

The FET-protein family includes FUS (fused in sarcoma, also called TLS (translocated in liposarcoma)), EWS (Ewing sarcoma breakpoint 1, also called EWSR1), and TAF15 (TATA box binding protein associated factor 68 kDa) [1]. The FET-proteins are RNA- and DNA-binding proteins composed of several conserved domains including a SYGQ-rich domain in the N-terminal part, a G-rich domain, an RNA-binding domain (RRM), a zinc-finger of RanBP2-type and a C-terminal RGG-rich domain [2]. The N-terminal domains have a transcriptional trans-activating function *in vitro* [3]. The RRM, zinc-finger, and RGG-rich domains are all involved in the RNA-binding of the FET-proteins [4], [5], [6], [7]. The FET-proteins associate with a number of factors involved in transcription and RNA processing such as RNA Polymerase II and splicing factors [1], [6], [8], [9], [10]. Moreover, FET-proteins are identified in the Drosha miRNA processing complex [11], [12]. The functions of the RNA-binding of FET-proteins are not completely elucidated, but FUS is recruited by non-coding RNAs to the cyclin D1 gene and inhibits the expression upon DNA-damage [13].

The FET-proteins are expressed in most human tissues and mainly localize to the cell nucleus [14], although they are able to shuttle between the nucleus and the cytoplasm [6], [15], [16]. FUS and EWS harbor nuclear localization signals in their C-terminus [17], [18], whereas TAF15 nuclear localization is controlled by arginine methylation in the RGG-rich domain [15]. Both FUS and EWS are detected in dendritic RNA-transporting granules suggesting a role in RNA transportation and localization [19], [20], [21]. During the mammalian brain development the FET proteins are expressed in an identical pattern in neurons and glial cells, and the expression declines throughout the brain development [22]. The FET-protein family is implicated in neurodegenerative diseases and cancer. The genes are fusion partners in a large number of cancer-associated translocations [23], [24] and are overexpressed in liposarcoma cell lines [25]. FUS is also overexpressed in prostate tumors [26]. Mutations in the FUS, EWS, and the TAF15 genes are reported in familiar and sporadic amyotrophic lateral sclerosis (ALS) [27], [28], [29], [30].

A hallmark of neurodegenerative diseases is the progressive accumulation of aggregates of misfolded proteins termed proteinopathies [31]. Induction of cellular stress can contribute to the protein aggregation [32]. Stress granules (SGs) are non-membranous cytoplasmic aggregates, comprised of non-translating messenger ribonucleoproteins. These structures form in cells that are exposed to environmental stress such as heat shock, oxidative stress, hyperosmolarity, viral infection or UV irradiation [33]. In response to stress, general translation is stalled at the level of initiation, which activates aggregation-prone proteins to sequester abortive 48S pre-initiation complexes and rapidly aggregate into SGs. The precise function of SGs is not entirely clear, and it has been suggested that SGs are sorting granules for mRNAs undergoing degradation, storage or translation. In line with this, SGs are highly dynamic structures that contain RNA-binding proteins, transcription factors, RNA helicases, nucleases, kinases, and signaling molecules [33]. FET-proteins are reported to relocate to SGs in response to oxidative stress [14], [17], [18], [34]. ALS associated FUS mutations lead to neuronal cytoplasmic FUS and ubiquitin positive inclusions and mutated FUS is more rapidly directed to SGs after oxidative stress than wild type FUS [17], [18], [34], [35]. FUS- and ubiquitin-positive inclusions are also found in a variant of frontotemporal lobar degeneration (FTLD), termed FTLD-FUS [36]. Moreover, a higher grade of cytoplasmic FUS localization is associated with an earlier onset and faster developing form of ALS. FUS and ubiquitin positive inclusions in ALS and FTLD-FUS also contain SG markers indicating that SG formation may be implicated in the generation of protein inclusions [18]. In ALS with FUS mutations no TAF15 and EWS inclusions are observed whereas in FTLD-FUS also TAF15 and, variably, EWS inclusions are present [37]. The FET-proteins are also identified in intracellular polyglutamine mediated protein aggregates observed in trinucleotide expansion diseases [38], [39], [40].

In this study we have examined the role of the FET-proteins in oxidative stress and SG formation. We have used the two human cell lines HEK293 and SH-SY5Y and found that both FUS and TAF15 localizes to SGs in a cell stress dependent manner whereas EWS SG localization was rarely observed. FET-protein reduction had no influence on SG formation suggesting that FET-proteins are not vital for SG formation, but mainly localize to SGs in a downstream response to cellular stress. Moreover, the localization of FUS and TAF15 in SGs is correlated with an observed higher similarity of the transcriptional regulatory functions between FUS and TAF15 compared with EWS.

## Results

### FUS and TAF15 Localize to Stress Granules more Consistently than EWS

**Figure 1:**
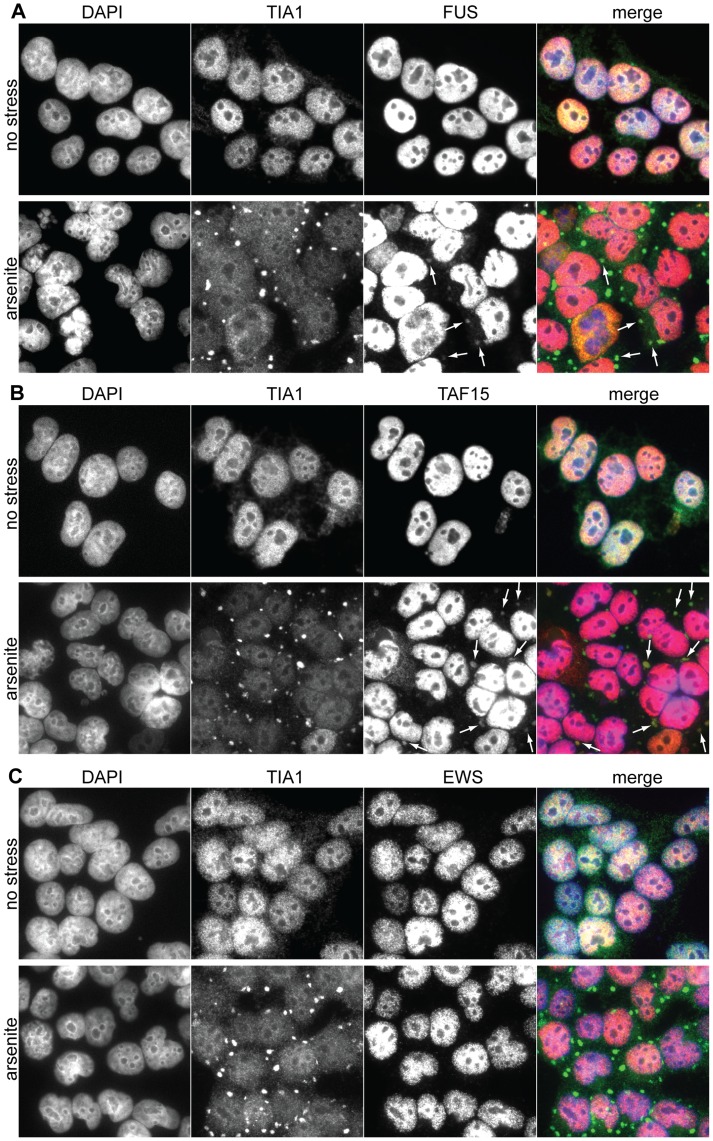
Immunostainings of the FET-proteins and TIA1 in HEK293 cells after arsenite stress. (**A**) TIA1 and FUS immunostaining. (**B**) TIA1 and TAF15 immunostaining. (**C**) TIA1 and EWS immunostaining. The nuclei are counterstained by DAPI.

FET-proteins were previously shown to localize to TIA1-positive SGs in response to arsenite, heat-shock, and inhibition of Transportin mediated nuclear import [14], [37]. In order to further examine the localization of the FET-proteins in response to stress we used the human cell line HEK293 derived from embryonic kidney cells with neuronal resemblance [41], [42]. HEK293 cells were treated with arsenite and immunostained for FUS, TAF15, and EWS (Figure 1A–C). To identify SGs, cells were co-stained for the SG marker protein TIA1 [33], [43]. The nucleus was counterstained with DAPI. In the merged picture in each panel the FET-proteins are shown in red, TIA1 in green and the nucleus is stained with DAPI (blue) (Figure 1). Without stress TAF15, FUS, and EWS localize to the nucleus (Figure 1A–C), but after arsenite-induced stress FUS (Figure 1A, indicated by white arrows) and TAF15 (Figure 1B, indicated by white arrows) are also detectable in TIA1-positive SGs (Figure 1A–B, merge). The major fraction of TAF15 and FUS is however still located in the nucleus. TAF15-positive SGs are visible in a majority of the cells and FUS positive SGs in approximately 20% of the stressed cells (Figure 1A). No EWS was observed to localize in SGs in the HEK293 cells (Figure 1C) and thereby it serves as an important negative control for potential SG-signal “bleeding” from the green to the red channel. The experiment was repeated with the human neuroblastoma cell-line SH-SY5Y (Figure S1) [44]. Also in SH-SY5Y cells TAF15 and FUS could be observed in TIA1-positive SGs. In less than 1% of the SH-SY5Y cells EWS was detected in SGs (Figure S1).

### FET-proteins are not Required for Stress Granule Formation

**Figure 2:**
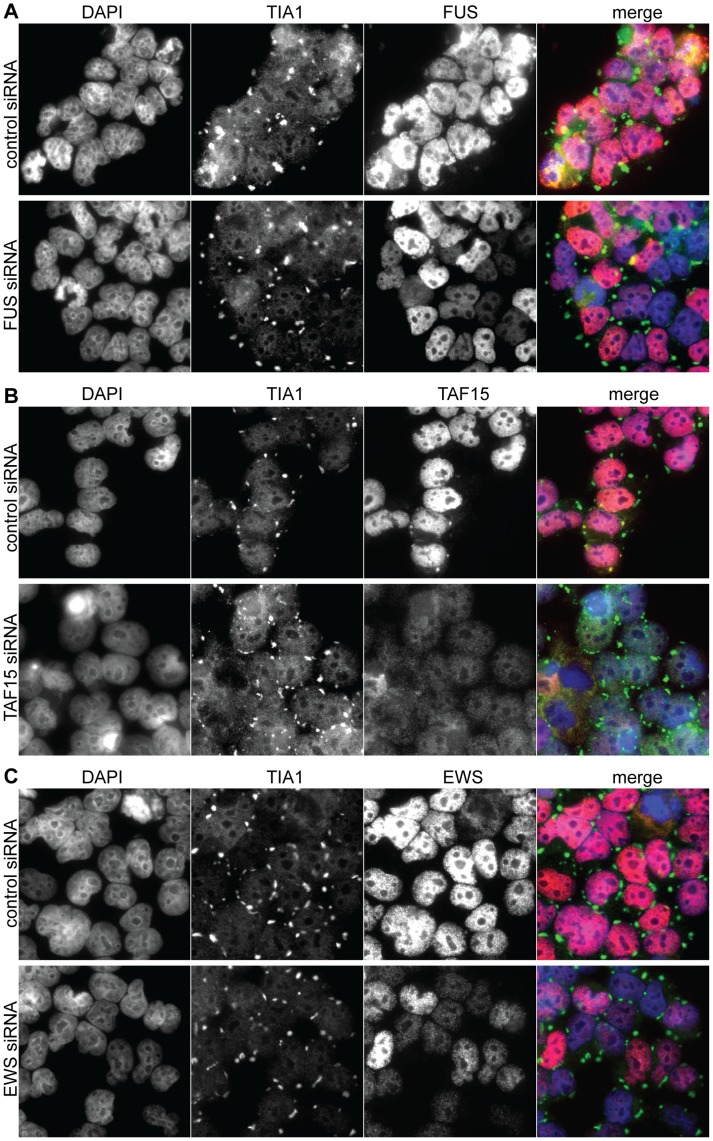
Immunostainings of the FET and TIA1 proteins in HEK293 cells after siRNA-mediated gene knock-down of the FET proteins together with arsenite induced stress. Control cells were transfected with an equal amount of siRNA with an unspecific sequence. (**A**) TIA1 and FUS immunostaining. The nuclei are counterstained by DAPI. (**B**) TIA1 and TAF15 immunostaining. The nuclei are counterstained by DAPI (**C**) TIA1 and EWS immunostaining. The nuclei are counterstained by DAPI.

To further clarify the roles of FET-proteins in SGs, we analyzed whether they are necessary for SG formation. HEK293 cells were transfected with siRNAs against FUS, TAF15, or EWS mRNAs, separately, all three in combination, or a control siRNA. Cells were stressed with arsenite and immunostained for FUS, TAF15, and EWS (Figure 2A–C). SGs were identified by co-staining for TIA1 (Figure 2A–C). The expression of FUS, TAF15, and EWS protein (red) is significantly reduced in the siRNA transfected cells, compared to the cells transfected with unspecific siRNA (Figure 2A–C, third column). After arsenite treatment of cells triple transfected with siFUS, siEWS, and siTAF15, TIA1-positive SGs (green) were still consistently detected at the same frequency as in control cells, indicating that normal amounts of FET-protein are dispensable for SG formation (Figure S2, Table S1).

**Figure 3:**
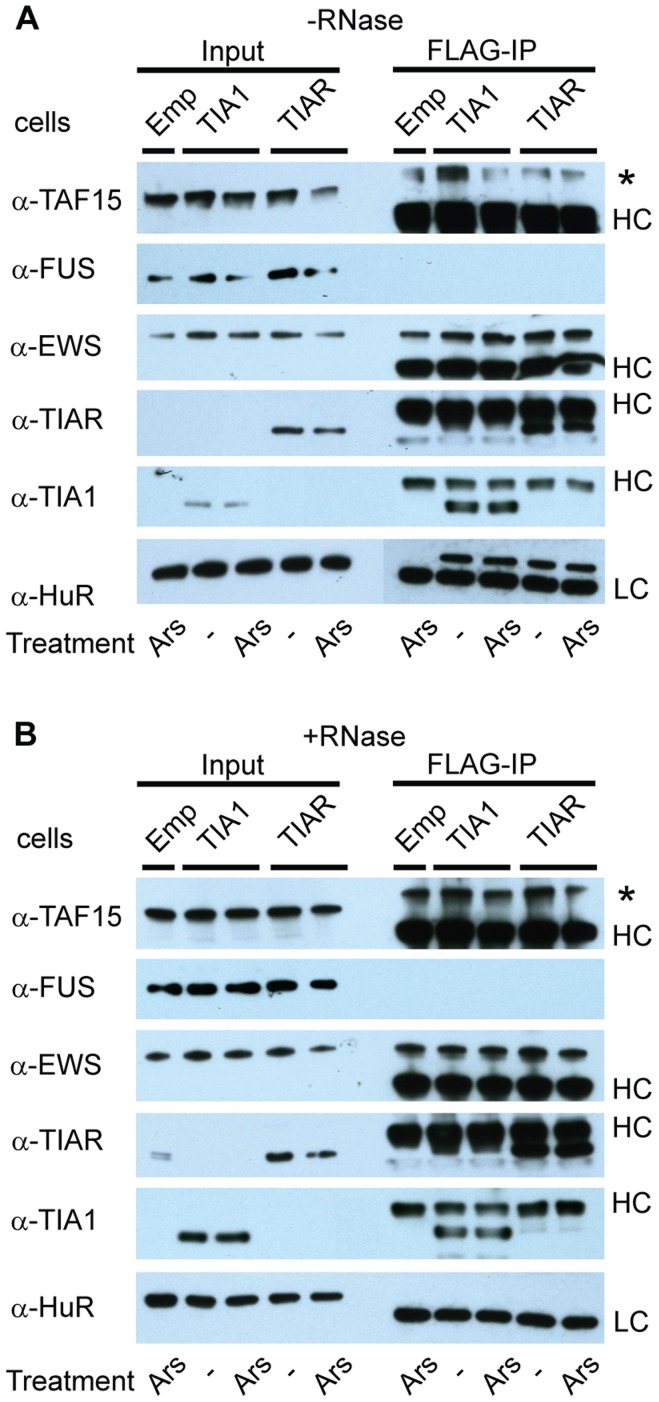
Western blot of co-immunoprecipitation of the FLAG-TIA1 and FLAG-TIAR proteins shows no significant binding to FUS, EWS, or TAF15. (**A**) Co-immunoprecipitation conducted without RNase A. The used FLAG cell line is shown above the blot. “Emp” is HEK293-cells without FLAG vector used as control. Unstressed control cells are marked (-), and stressed arsenite treated cells (Ars). (*) marks an unidentified background protein, and HC and LC is heavy and light chain, respectively, from the used mouse antibodies. HuR is used as control protein for an intact RNA dependent interaction to TIA1 and TIAR. (**B**) The co-immunoprecipitation conducted with RNase A. (*) marks an unidentified background protein, and HC and LC is heavy and light chain respectively from the used mouse antibodies.

The localization of both FUS and TAF15 to SGs prompted us to test whether the FET-proteins form a complex with SG-factors. To address this question, we performed co-immunoprecipitation assays using HEK293 cell lines stably expressing FLAG-tagged TIA1, TIAR or as a control, an HEK293 cell line lacking an expression cassette (marked “Emp”) (Figure 3A). Neither FLAG-TIA1 nor FLAG-TIAR detectably co-purified with FUS, TAF15 or EWS, even during arsenite-induced oxidative stress (Figure 3A). A repeated experiment with omission of RNAse A also failed to detect an RNA-dependent interaction between the TIA1 and TIAR proteins and TAF15, EWS or FUS (Figure 3B). We conclude that under the experimental conditions used, we were unable to detect a robust complex between the TIA1 and TIAR proteins and TAF15, EWS or FUS.

### The FET-mRNA Levels Respond Differentially to Oxidative Stress

**Figure 4:**
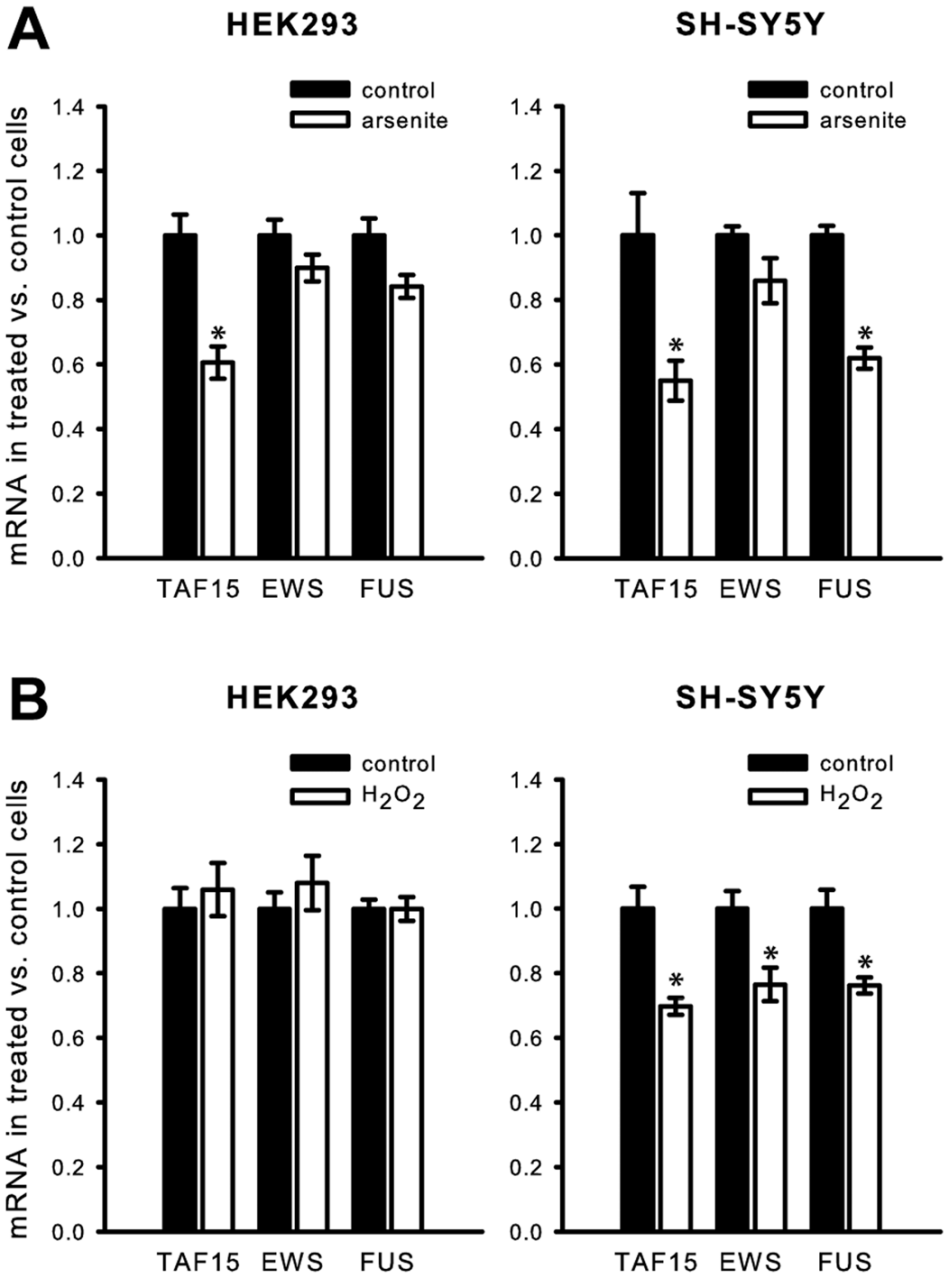
RT-qPCR of the FET mRNA expression in HEK293 and SH-SY5Y cells after oxidative stress. The FET mRNA levels were normalized to the expression of GAPDH and quantified to the FET mRNA level in unstressed cells according to the X_0_-method [45]. (**A**) Arsenite induced cellular stress. *, P<0.001. **, P<0.01. (**B**) H_2_O_2_ induced cellular stress. *, P<0.005. **, P<0.01.

To further analyze the function of the FET-proteins in response to oxidative stress, we analyzed the FET mRNA expression in HEK293 and SH-SY5Y cells treated with either arsenite or H_2_O_2_ (Figure 4). RNA was extracted from arsenite or H_2_O_2_ treated cells and the FET mRNA expression was measured by RT-qPCR. The FET mRNA levels were normalized to the expression of GAPDH and quantified according to the X_0_-method [45]. The expression of TAF15 mRNA was decreased by 40% in both HEK293 and SH-SY5Y cells after arsenite-treatment (Figure 4A). The FUS mRNA level was also decreased by 40% in SH-SY5Y but not as robustly in HEK293 cells (Figure 4A). The EWS mRNA expression was not significantly affected by arsenite treatment in either cell line. Thus, FET-mRNAs have a different response to arsenite-induced stress. On the contrary, H_2_O_2_ treatment, did not significantly alter the expression of any of the FET-mRNAs in HEK293 cells (Figure 4B left), while all were reduced by approximately 25% in SH-SY5Y cells (Figure 4B right). We did not detect any corresponding change of FET-protein expression after identical oxidative stress induction by H_2_O_2_ or arsenite (Figure S3). The incubation times for the induction of oxidative stress by H_2_O_2_ and arsenite were 2 h and 1 h, respectively. That the observed reductions in mRNA expression are not detected at the protein level could be due to the stability of the FET-proteins.

### The FET-proteins have a Minimal Impact on Oxidative Stress Induction

**Figure 5:**
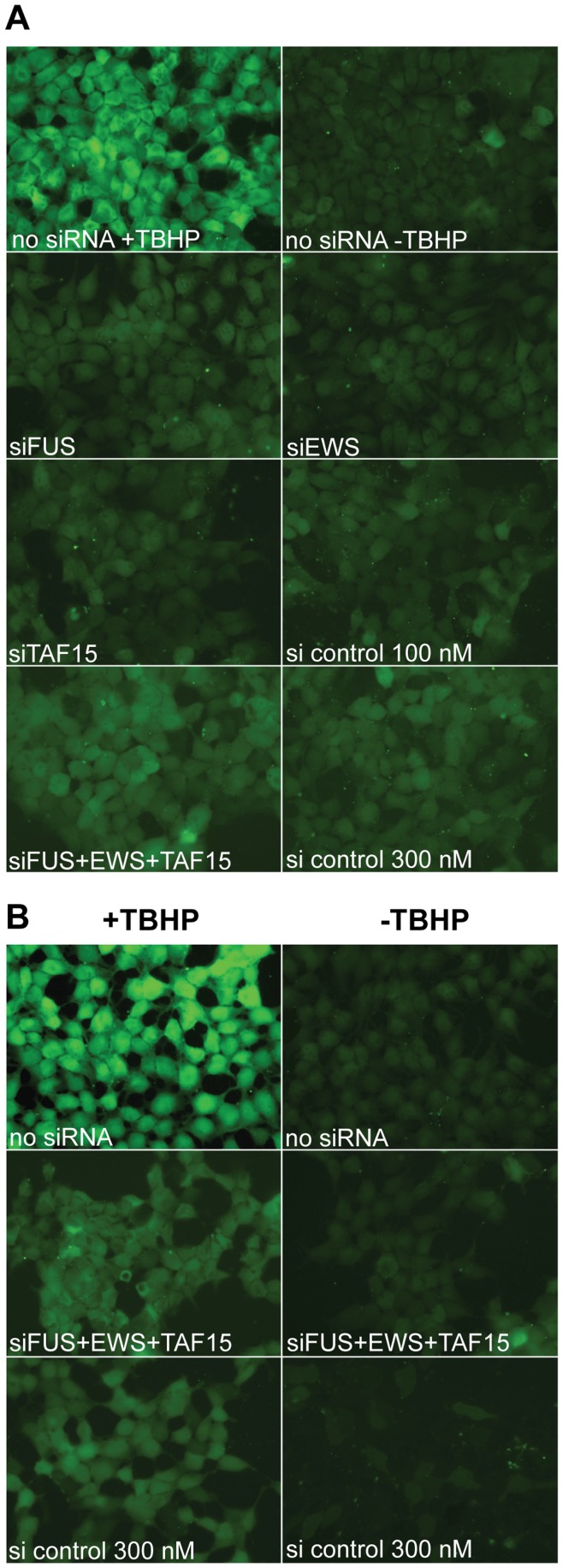
FET-protein reduction is not affecting ROS. (**A**) HEK293 cells were transfected with siRNAs targeted against the FUS, EWS, or TAF15 mRNAs, or with an unspecific siRNA as control. Cells were also transfected with a combination of all three FET siRNAs (300 nM total concentration) or unspecific siRNA at concentration of 300 nM (lower panel). The level of ROS in the cells was measured and evaluated by microscopy. Untransfected cells treated with TBHP is a positive control of oxidative stress (upper panel left), and untransfected cells without TBHP treatment is a negative control of oxidative stress (upper panel right). (**B**) HEK293 cells were transfected with a combination of all three FET siRNAs (300 nM total) or with an unspecific siRNA as control. Cells were subsequently treated with TBHP to induce oxidative stress (left columns), and the level of ROS was measured and evaluated by microscopy and compared to unstressed cells (right columns). Untransfected cells treated with TBHP is a positive control of oxidative stress (upper panel left), and untransfected cells without TBHP treatment is a negative control of oxidative stress (upper panel right).

The role of the FET-proteins in oxidative stress was further analyzed. A putative function of the FET-proteins could be to hinder oxidative stress by mediating a reduction in the level of reactive oxygen species (ROS) present in the cell. To test this, HEK293 cells were transfected with siRNAs against TAF15, EWS, and FUS, and the levels of ROS visualized by 5-(and-6)-carboxy-2′,7′-dichlorodihydrofluorescein di-acetate (carboxy-H_2_DCFDA) as a fluorogenic marker for ROS in living cells. After treatment, cells were mounted and immediately photographed and quantified for pixel intensity (Figure 5A and Table S2). Non-transfected cells treated with tert-butyl hydroperoxide (TBHP) to induce ROS production are positive controls for oxidative stress (Figure 5A, panels no siRNA +/− TBHP). siRNA against FUS, EWS or TAF15 did not result in increased levels of ROS compared to siRNA control transfected cells (Figure 5A). In cells transfected with a combination of FUS+EWS+TAF15 siRNA the ROS level increased modestly, but insignificantly, compared to the siRNA control cells (Figure 5A and Table S2).

We next analyzed how HEK293 cells respond to oxidative stress after knockdown of the FET-proteins. Cells were transfected with a combination of FUS+EWS+TAF15 siRNAs and treated with TBHP to induce oxidative stress. The level of ROS was measured by microscopy and pixel counting and compared to unstressed cells (Figure 5B and Table S2). Non-transfected cells treated with TBHP serve as positive controls for oxidative stress. Although, the measured ROS levels after TBHP treatment was lower in the FET siRNA transfected cells compared to the positive control (Figure 5B), a similar decrease in ROS production was also observed in the control cells (Figure 5B). Thus, reduction of FET-proteins has no detectable influence on the level of ROS under the given assay conditions.

### Reduction of FET-proteins Effect Cellular Gene Expression Profiles Differently

**Figure 6:**
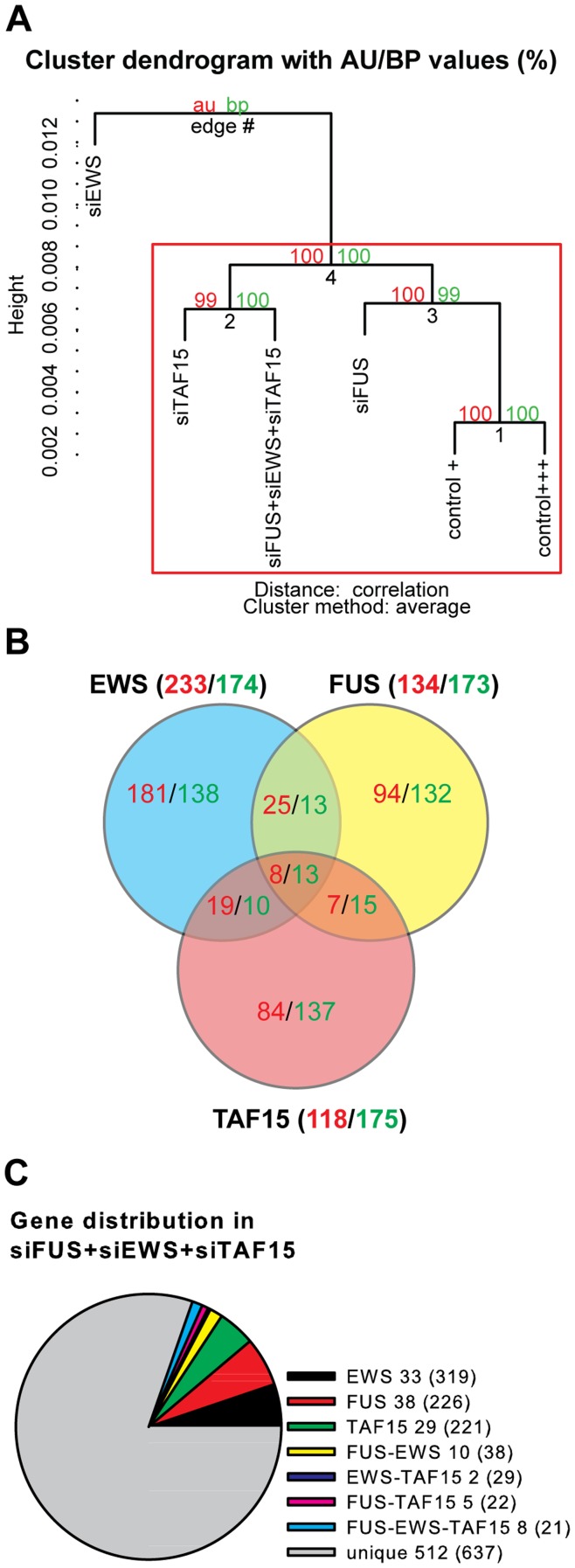
Analysis of the Illumina HumanGW 6 BeadChip whole genome expression array. (**A**) Hierarchical clustering by Pvclust by of the significantly expressed genes in HEK293-cells transfected with siRNAs for the FET mRNAs (siFUS, siEWS, siTAF15, siFUS+siEWS+siFUS) or an unspecific control siRNA (control + and control +++). The control + is control sample for the individual FET siRNA transfections, and the control +++ for the siFUS+siEWS+siTAF15 sample, to ensure the comparisons of equal siRNA concentrations. P-values (%) are shown on the edges of the clustering, Approximately Unbiased (AU) p-values in red, and Bootstrap Probability (BP) values in green. Clusters with an AU p-value greater than 95% are highlighted by a red rectangle. (**B**) The distribution of differentially expressed genes (DEGs) in the siFUS (yellow), siEWS (blue), and siTAF15 (light red) transfected cells. The up-regulated number of genes is shown in red and down-regulated in green. The number of common DEGs is shown in the overlapping parts of the circles. (**C**) DEGs in the siFUS, siEWS, and siTAF15 transfected cells. The common DEGs were compared to the DEGs in the siFUS+siEWS+siTAF15 transfected cells. In total 637 DEGs are identified in the siFUS+siEWS+siTAF15 cells, of those are 512 unique genes (grey). 33 of the 319 common DEGs in siEWS transfected cells were found (black), 38 of 226 in FUS (red), 29 of 221 in TAF15 (green), 10 of 38 in FUS and EWS (yellow), 2 of 29 in EWS and TAF15 (dark blue), 5 of 22 in FUS and TAF15 (pink), and 8 of 21 in FUS and EWS and TAF15 were found (blue).

**Figure 7:**
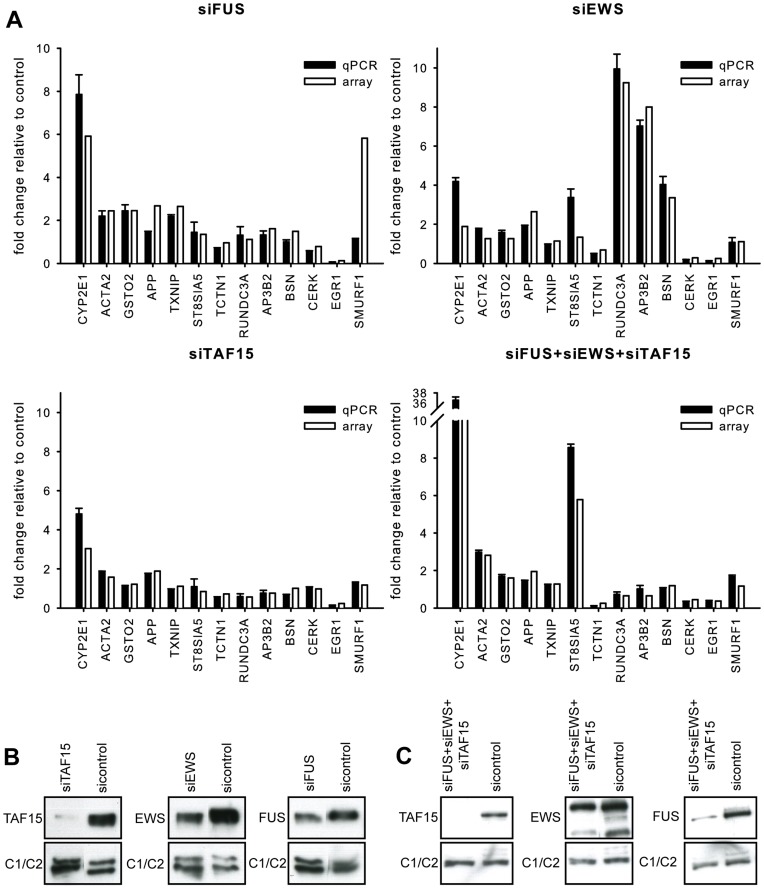
Verification of the gene expression array analysis. (**A**) RT-qPCR analysis of siFUS, siEWS, siTAF15, and siFUS+siEWS+siTAF15 transfected HEK293 cells. 13 candidate genes which expression was altered in the array analysis were chosen. RT-qPCR was performed in triplicates, and the gene expression was normalized to the expression of the housekeeping gene GAPDH [45]. The expression level as measured by RT-qPCR is shown in black, and the measured level in the array is shown in white. Standard deviations from the technical triplicates are shown. (**B**) FUS, EWS, and TAF15 protein expression in the siFUS, siEWS, and siTAF15 transfected HEK293 cells used in the expression analysis. Control cells are transfected with an equal amount of unspecific siRNA. The protein expression of the ubiquitously expressed protein hnRNPC1/C2 (heterogeneous nuclear ribonucleoprotein C (C1/C2)) is shown as loading control. (**C**) FUS, EWS, and TAF15 protein expression in the siFUS+siEWS+siTAF15 transfected HEK293 cells used in the expression analysis. Control cells are transfected with an equal amount of unspecific siRNA. The protein expression of hnRNPC1/C2 is shown as loading control.

The FET-proteins are putative transcriptional regulators and, given the above described differences in FET protein sequestration in SGs in response to cellular stress, we next analyzed if FUS, EWS, and TAF15 are part of similar or distinct transcriptional regulatory pathways by microarray analysis (Figure 6). For this purpose HEK293 cells were transfected with siRNAs directed against FUS, EWS, and TAF15, or with a combination of the three siRNAs. For FUS and EWS siRNA pools predesigned by Dharmacon consisting of 4 siRNAs each targeting the mRNA but at separate locations were used. For TAF15 a mix of two siRNAs was used. One siRNA was designed using the webbased siDESIGN center by Dharmacon and the other is identical to the one used in the study of Jobert et al [15]. Control cells were transfected with equal amounts of a non-specific siRNA used in the study of Zhou et al [46]. The siRNA transfection reduced the FET-mRNA levels as well as the protein levels, and the siRNAs showed specificity for the target FET protein as well as mRNA (Figure 7, and data not shown). Total RNA from transfected HEK293 cells was extracted and the samples were analyzed using one Illumina HumanGW 6 BeadChip. HEK293 cells were transfected in duplicate samples which were pooled after lysis to generate one mRNA sample for each subtype of siRNA treatment (siTAF15, siEWS, siFUS, siControl, siFUS+siEWS+siTAF15, and siControl for the triple transfection). The Illumina HumanGW 6 BeadChip contains 6 separate arrays and each mRNA sample was analysed by one array. The Raw data are deposited in the NCBI’s Gene Expression Omnibus [47] and are accessible through GEO Series accession number GSE35578 (http://www.ncbi.nlm.nih.gov/geo/query/acc.cgi?acc=GSE35578). The raw data were normalized with quartile normalization, followed by filtering to remove non-significantly expressed mRNA (22059 out of total 48803 probes removed). Moreover, background intensity was subtracted from the gene expression intensity. The significantly expressed genes in each group were hierarchically clustered using Pvclust, to analyze how similar the gene expression is between the siRNA groups [48]. To lower the “noise” in the analysis, the Pvclust analysis disregards from very similarly expressed genes in all samples, a selection based upon the standard deviation. Thereby an analysis of only the variation in the overall gene expression between the samples is made. Pvclust generates two types of p-values, Approximately Unbiased (AU) and Bootstrap Probability (BP). The clustering of the gene expression in siFUS, siEWS, siTAF15, and siFUS+siEWS+siTAF15 siRNA transfected cells, and control cells are shown in Figure 6A. P-values (%) are shown on the edges of the clustering, AU p-values in red and BP values in green. Clusters with an AU p-value greater than 95% are highlighted by a red rectangle. The clustering shows that gene expression profiles in siTAF15, siFUS, siFUS+siEWS+siTAF15, and control cells are more similar to each other than to siEWS cells (Figure 6A).

Next we quantified the mRNA levels in siRNA transfected cells relative to control cells. Gene expression was defined to be up-regulated if the ratio of the expression intensity between the siRNA transfected cells and the control cells was larger than 2 and the detection p-value of gene expression in siRNA treatment group was below 0.05. Similarly, the gene expression was considerable down-regulated if the ratio between the siRNA transfected cells and the control cells was below 0.5 and the p-value was below 0.05. The output of this selection procedure is defined as the differentially expressed genes (DEGs). The distribution of DEGs in the siFUS (yellow circle), siEWS (blue circle), and siTAF15 (light red circle) is shown in Figure 6B. The number of up-regulated DEGs is shown in red, down-regulated DEGs in green and the common DEGs in the three groups are annotated in the overlays of the individual circles. In total 307, 293, and 407 DEGs were found with siFUS, siTAF15 and siEWS, respectively (Figure 6B). In siFUS and siTAF15 fewer genes were up-regulated (134 and 118 respectively) than down-regulated (173 and 175 respectively). On the contrary, in siEWS more genes were up-regulated than down-regulated (233 and 174 respectively). 21 DEGs were common for all the three groups (the center overlay of all the circles), and the commonly identified DEGs are listed in Table S3. Commonly identified DEGs for siFUS+siEWS are 59 in total, and for siFUS+siTAF15 43. The higher number of commonly identified DEGs in siFUS+siEWS may suggest that FUS and EWS share closer resemblance, and is different from the results from the Pvclust analysis displayed in Figure 6A. However, note that we define a DEG as a larger than 2-fold change compared to the control. In the Pvclust analysis in Figure 6A a higher number of genes are included. Thus, when considering the overall expressed genes, FUS and TAF15 are closer related than each other than either of them to EWS. When only considering the DEGs, EWS and FUS are closest related to each other. The DEGs from Figure 6b were compared to the DEGs in siFUS+siEWS+siTAF15 (Figure 6C). Most of the DEGs in siFUS+siEWS+siTAF15 (512 out of 637) are unique for this group, and not significant in the cells transfected with the single FET siRNAs, suggesting an additive effect.

The 10 most up- and down-regulated genes in siFUS-, siEWS-, siTAF15-, and siFUS+EWS+TAF15-transfected cells are summarized in Table S4 and Table S5 and the complete list of DEGs in Table S6. By RT-qPCR we validated the results for a fraction of the identified DEGs (Figure 7). One example is the gene CYP2E1 (cytochrome P450, family 2, subfamily E, polypeptide 1), which in the array analysis is up-regulated 17-fold in the siFUS+siEWS+siTAF15 cells and up-regulated 6-, 3-, and 1.8-fold in siFUS, siTAF15, and siEWS cells, respectively. Concordant data was observed by RT-qPCR (Figure 7). By over-expressing FLAG-tagged FET proteins FUS, EWS, and TAF15 proteins in HEK293-cells we could for FUS (3 of 6), EWS (3 of 8), and TAF15 (1 of 3) by RT-qPCR detect the opposite regulation than observed in siRNA transfection experiments (Figure S4). In addition, RT-qPCR analysis showed that the changes in gene expression observed in HEK293 cells for most of the tested genes could be confirmed in material from siRNA transfected SH-SY5Y neuroblastoma cells (Figure S5). We analyzed if the CYP2E1 protein is correspondingly up-regulated after siRNA transfection of HEK293-cells, but could not detect any expression at all of the CYP2E1 protein in HEK293-cells (Figure S6a–c). The DEGs in the siFUS, siEWS, siTAF15, and siFUS+siEWS+siTAF15 samples were subjected to gene ontology analysis using the web-based program Ingenuity IPA 9.0 (Figure S7, Table S7, Table S8, Table S9, and Table S10). siEWS differed from siFUS and siTAF15 in that the category “neurological disease” was among the most significantly detected pathways and deregulation of a gene subset in this pathway was verified by qPCR (Figure 7).

## Discussion

We show that a fraction of TAF15 and FUS co-localizes with TIA1 in SGs after oxidative stress in HEK293 and SH-SY5Y cells. In HEK293 cells TAF15 localization to SGs is detected in the majority of the cells and FUS in 20% of the cells after arsenite-treatment. In SH-SY5Y cells FUS and TAF15 are also identified in SGs, but at a lower frequency than in HEK293 cells. We could only detect EWS in SGs in very few SH-SY5Y cells and not at all in HEK293 cells. The localization of all three FET-proteins to SGs was reported previously [14]. Notably, an EWS-GFP fusion protein was rarely located to SGs whereas endogenous EWS was designated a localization to SGs [14]. Other reports have shown that FUS cannot be identified in SGs [17], [18], [34]. In all these other studies confocal microscopy was used, while we use wide-field microscopy. Since both positive and negative FUS SG localization is observed with confocal microscopy, we argue that the identification of FUS in SGs in the present study is not due to the used microscopy technique. There may be a possibility that EWS localization in SGs is better detected using confocal microscopy. The controversies about whether individual FET-proteins are located in SGs could be due to variations in the level of induced stress. We also treated HEK293 and SH-SY5Y cells with H_2_O_2_ for a milder form of oxidative stress than arsenite. By this procedure the presence of TAF15 and FUS in HEK293 SGs was rarer and in SH-SY5Y cells FUS could not be detected in SGs. Taken together these observations suggest that the redirection of the FET-proteins to SGs depends on a certain threshold stress level and that TAF15, FUS, and EWS respond differently to cellular stress levels by SG association. In this context it is notably that blocking of Transportin specific nuclear import results in a more efficient recruitment of FUS and TAF15 into stress granules compared with EWS [37].

After FET protein reduction by siRNA mediated gene knock-down, TIA1 positive SGs are still detected at the same frequency. This indicates that expression of the FET-proteins at the normal cellular level is not necessary for the formation of SGs. TDP-43 (TAR DNA binding protein 43) has resemblance to the FET-proteins and TDP-43 co-localizes with TIA1 in SGs [49]. A study reported that TDP-43 contributes to the assembly and maintenance of SGs [50], whereas another study is contradictory [49]. These two studies use different arsenite incubation times, 15–30 min, and 1 h respectively. ALS-causing mutations have been identified in TDP-43, FUS, EWS and TAF15 [28], [30], [51], [52]. TDP-43 and FUS are identified in cytoplasmic aggregates resembling SGs in FTLD brains [18], [36], [49]. The presence of TDP-43, FUS, and TAF15 in SGs may be a common link to their involvement in ALS and FTLD. FUS and TDP-43 with ALS mutations locate to a much greater extent to the cytoplasm and are directed faster to SGs after oxidative stress [17], [18], [34], [49]. A higher degree of cytoplasmic re-localization of mutated FUS correlates with a younger age of onset and faster ALS development [18]. The expression of TAF15 mRNA is reduced by 40% in HEK293 and SH-SY5Y cells after oxidative stress induced by arsenite. In addition, after arsenite treatment, the FUS mRNA expression is also decreased by 40% in SH-SY5Y cells. By H_2_O_2_ treatment the expression levels of the FUS, EWS, and TAF15 mRNAs were moderately reduced in SH-SY5Y cells but unaffected in HEK293 cells. It is questionable if the FUS and TAF15 mRNA levels decrease as a result of the oxidative cell damage, or the reduction is mechanistically involved in protecting cells from oxidative stress. We did not observe increased oxidative stress after siRNA mediated reduction of FET-protein expression and induced oxidative stress produces equal amounts of ROS in FET siRNA treated cells and control cells. This indicates that FET proteins do not influence the level of cellular ROS. We find it more likely that the reduction in FUS and TAF15 mRNA level caused by arsenite treatment is a result of the general oxidative damage. All in all our observations together with previous reports strongly indicate that the TAF15 and FUS response towards cellular stress have common characteristics, which differs from the EWS response.

In order to investigate if the gene regulatory functions of the three FET-proteins were similar, we conducted an expression array analysis of RNA from FET-protein depleted HEK293 cells. We note that the use of a single array for each mRNA sample instead of array triplicates with biological replicates increase the probability that individual genes can be scored as false positives. One of the identified highly up-regulated DEGs is CYP2E1 and we note that the altered CYP2E1 expression also was observed by qPCR in biological replicates in HEK293 cells as well as in SH-SY5Y cells (Figure 7 and data not shown). The CYP2E1 protein is expressed in the liver where it oxidizes more than 80 xenobiotics [53] and ethanol is a major substrate. CYP2E1 protein is also expressed in the human brain substantia nigra [54]. Increased ROS production is observed in the substantia nigra when substrates for CYP2E1 are present [54], and a mutation in the CYP2E1 gene is associated with Parkinson’s disease [55]. One substrate for CYP2E1 is formaldehyde which is oxidized to formic acid [56]. There is an association between the risk of developing ALS and formaldehyde exposure [57], as well as between risk of ALS and smoking [58]. Despite the fact we could not detect any altered expression of the CYP2E1 protein expression in HEK293-cells, hypothetically, defects in FET-proteins could increase CYP2E1 expression in the brain, which may cause higher rates of oxidative stress in brains of exposed individuals and thus faster development of ALS. Further investigations of the implications of the described findings will be required to clarify the relation between FET-proteins, stress responses, and neurodegenerative diseases.

Clustering of significantly expressed genes showed a distinct gene expression profile in siEWS transfected cells compared to the siFUS and siTAF15 transfected cells. Reduction of EWS resulted in a proportionally higher number of up-regulated than down-regulated genes, whereas reduction of FUS and TAF15 lead to more down-regulated than up-regulated genes. These analyses suggest a closer resemblance of TAF15 and FUS to each other, compared to EWS. Pathway analysis by Ingenuity IPA 9.0 showed that commonly identified pathways in siFUS, siEWS, and siTAF15 cells are regulating cell death, cellular development, and cell morphology. Recently, FUS was identified to interact with PGC-1α (peroxisome proliferator activated receptor γ coactivator 1α), and together they regulate the transcription of oxidative stress protection genes in rat liver cells [59]. We conducted an oxidative stress assay, but no alteration in ROS content upon FET-protein reduction was detected. Taken together with the fact that FUS and TAF15 localize to SGs indicates that they are involved in the general oxidative damage response of cells. Further research will be required to confirm and delineate the involvement of the FET-proteins in cellular signal transduction pathways related to oxidative stress response.

After this study was completed a study was presented containing an identification of the RNA targets for the FUS, EWS, and TAF15 proteins [60]. We compared the genes identified as FET RNA targets by Hoell et al. with the DEGS identified in this study. 70 out of the 307 DEGs for FUS, 75 out of 407 DEGs for EWS, and 30 out of 293 DEGs for TAF15 are identified, but this correlation is not statistically significant (Table S11). Further research will be required to identify the gene regulatory mechanisms of the FET-proteins.

## Materials and Methods

### Cell Culture and Growth Conditions

HEK293 cells and SH-SY5Y cells were grown in D-MEM with 10% Fetal Bovine Serum, streptomycin (2.0 g/l), penicillin (1.2 g/l) and glutamine (0.3 g/l) (complete D-MEM) at 37°C and 5% CO_2_. Cells were propagated with the use of Trypsin solution 0.05% EDTA (Gibco). HEK293 and SH-SY5Y-cells were obtained from the American Type Culture Collection (CRL-1573 and CRL-2266, respectively).

### siRNA-mediated Gene Knock-down of the FET-proteins

Cells were transfected using Dharmafect 1 (Dharmacon) according to manufacturer’s instructions. Briefly, 24 h before transfections cells were reseeded in fresh media at 40% confluence. At the day for transfection cells were trypsinized and counted. For HEK293 cells, 100000 cells were used in a 24-well plate with 100 nM final siRNA concentration or 300 nM for triple transfections. Transfections for RNA or protein extractions were performed in duplicates, and pooled after lysis. Cells were incubated for 72 h before analyzed. For SH-SY5Y cells, 200000 cells were used in a 24-well plate at a 200 nM final siRNA concentration. After 48 hours cells were transfected again with 200 nM final siRNA concentration using TransIT-siQUEST Transfection Reagent (Mirus). After another 48 hours cells were harvested. When transfecting cells for microscopy, only 50000 cells were used and plated on 17 mm poly-L-lysine coated glass slides in a 12-well dish. Predesigned pools of 4 siRNAs (ON-TARGET plus SMARTpool, Dharmacon) were used for FUS and EWS. Sequences for EWS: GAGUAGCUAUGGUCAACAA, GCAGAGAUCGGCCCUACUA, GAUCUAGGCCCUCCUGUAG, GCACUCAGCCUGCUUAUCC, and for FUS: GAUCAAUCCUCCAUGAGUA, GGACAGCAGAGUUACAGUG, GGACAGCAGCAAAGCUAUA, GAGCAGCUAUUCUUCUUAU. For TAF15 a mix of two siRNAs was used, one designed using the siDESIGN center by Dharmacon with the sequence UGAUCAGCGCAACCGACCATT, the other is identical to the one used in the study of Jobert et al [15] with the sequence UGAUCAGCGCAACCGACCA. Control cells were transfected with equal amounts of a non-specific siRNA used in the study of Zhou et al [46] with the sequence AGGUAGUGUAAUCGCCUUGTT.

### FET-protein Overexpression

Construction of FLAG-FET plasmids: Total RNA was extracted from human SW13-C1-cells, and cDNA made using Superscript III Reverse Transcriptase (Invitrogen). The open reading frames for the FUS, EWS, and TAF15 proteins were amplified by PCR using Phusion DNA Polymerase (Finnzymes). Forward and reverse primers included sequences for the restriction enzyme XhoI. The fragments were cut and ligated into the pSG5-expression vector containing a FLAG-tag immediately preceding the ORF. The sequences were verified by sequencing. Primer sequences are available upon request. 24 h before transfection, HEK293 cells (1×10^5^) were plated in each well of a 6-well dish. Cells were transfected in duplicates using FuGENE6 (Roche) according to the manufacturer’s instructions. 1 µg FLAG-FET plasmid was used in each well, and 0.2 µg EGFP-plasmid was added to measure transfection efficiency (above 80%). Control cells were transfected with 1.2 µg EGFP-plasmid alone. Cells were harvested for RNA and protein analysis 48 hours after transfection.

### RNA Extraction and cDNA Synthesis

RNA was extracted using TRI-Reagent (Sigma) according to the manufacturer’s protocol. cDNA was synthesized from 1 µg total RNA in 20 µl reactions using iScript™ cDNA synthesis Kit (Biorad). After synthesis, the cDNA was diluted five times with double distilled water and stored at −20°C.

### Protein Extraction

Cells were scraped off in Phosphate buffered Saline (PBS), and washed twice in PBS. Between washes cells were centrifuged at 1200 rpm 3 min between washes. Cells were added lysis buffer (50 mM Tris, 10 mM EDTA, 1% SDS, protease inhibitors (Complet Mini, Roche)) and lysed by three rounds of freezing at dry ice and thawing at 37°C. Cells were vortexed between each round. After lysis cells were centrifuged for 10 min 4°C at 14000 rpm, and the supernatant was saved. The protein concentration was measured by Bradford, and 0.4 µg protein was used for western blotting.

### Induction of Oxidative Stress and Stress Granule Formation

24 h prior to stress induction 200.000 cells (HEK293 or SH-SY5Y cells) were seeded in complete D-MEM onto poly-L-lysine coated slide glass in a 12-well dish. To induce oxidative stress and stress-granule formation, sodium arsenite (Arsenite) (Fluka, Sigma-aldrich) was used. Media was removed and D-MEM with 0.5 mM sodium arsenite (Fluka, Sigma-aldrich) was added to the cells. Cells were incubated in 37°C 1 h before continuing with the immunostaining procedure. For RNA extraction of oxidative stress induced cells, the cells were treated as above or with H_2_O_2_ (Sigma-Aldrich) but seeded directly into the 12-well dish and not onto slide glass. RNA was extracted as described above. For H_2_O_2_ stress induction the cell medium was removed and cells washed twice with PBS. D-MEM containing penicillin, streptomycin, and glutamine but without Fetal Bovine Serum, and including 600 µM H_2_O_2_, was added to the cells. Cells were incubated in 37°C 2 h before RNA extraction.

### Immunostaining

HEK293 cells or SH-SY5Y cells were grown on glass slides in 12-well cell culture dishes in complete D-MEM medium until 80% confluence. Cells were washed twice in PBS and fixed in 4% formaldehyde 15 min. Slides were washed twice in PBS, treated with 0,5% Triton X-100 in PBS for 10 min, and blocked in 1% BSA 1 h. The primary antibodies used were mouse anti TAF15 clone 8TA2B10 (a kind gift from Dr. Laszlo Tora), mouse anti EWS (sc-48404 Santa Cruz), and mouse anti FUS (sc-47711 Santa Cruz), all in 1∶200 dilutions. The cells were co-stained with a polyclonal goat antibody for the stress granule marker protein TIA1 (sc-1751 Santa Cruz) in a 1∶500 dilution, and incubated for 1 h at 37°C. Slides were washed three times in PBSM and incubated with secondary antibodies donkey anti-mouse Alexa Flour 555 (A31570 Invitrogen) and donkey anti-goat Alexa Flour 488 (A11055 Invitrogen) in a dilution of 1∶5000 incubation for 45 min at 37°C. Slides were washed 5 times in PBS containing 5 mM MgCl_2_, the nuclei counterstained with DAPI, slides washed twice in buffer and dried and mounted with ProLong Gold mounting media (Invitrogen). A Zeiss Axiovert 200 M microscope with CoolSNAP HQ camera was used. Pictures were processed using the ImageJ software.

### FET Depletion and Stress-granule Formation

HEK293-cells were transiently transfected with FET and control siRNA as described above. 72 h after transfection cells were stressed with sodium arsenite and immunostained as described above. Cells were randomly chosen on the slide and photographed using five different locations. The images of SGs were analyzed and the SGs counted using the ImageJ software. The counted SGs in each frame were then divided by the total number of cells in that frame to calculate an average SG content per cell. This was done for both FET and control siRNA, and the average SG number were compared.

### Oxidative Stress Assays

To analyze the cells for oxidative stress, the ROS-assay kit ImageIT (I36007) (Invitrogen) was used according to manufacturer’s instructions. HEK293 cells were transfected with siRNA as described above. 72 h after transfection, the growth medium was removed and cells were given fresh complete D-MEM containing 100 µM Tert-butyl hydroperoxide (TBHP). Cells were incubated in 37°C for 110 min. Pictures were taken with a Leitz DMRB microscope and a Leica DFC360FX camera using the Leica application Suite V3 software.

### Immunoprecipitation of TIA1 and TIAR Complexes

For immunoprecipitation of FLAG-tagged TIA1 and TIAR, HEK293S stable cell lines were grown in 10-cm plates to approximately 50% confluency and the expression of FLAG-tagged TIA1 and TIAR were induced by addition of 60 ng/ml tetracycline 36 hours prior to harvest. Cells were washed in cold PBS and lysed for 5 minutes on ice in 900 µl hypotonic lysis buffer (10 mM Tris-HCl pH 7.4, 10 mM NaCl, 2 mM EDTA, 0.1% Triton X-100, and either 0.5 mM PMSF, 2 µg/ml Aprotinin, 2 µg/ml Leupeptin or Complete Mini EDTA-free, Roche). The NaCl concentration was subsequently re-adjusted to 100 mM. Where indicated 5 µl RNase A (10 mg/mL) were added to the extracts to remove RNA. Lysates were cleared by centrifugation at 20,000×g and 800 µl supernatant was loaded onto pre-equilibrated anti-FLAG M2 agarose (Sigma, 20 µl bead volume) and incubated at 4°C for 3 hours. After a total of 8×1 ml washes in NET-2 buffer (50 mM Tris-HCl, pH 7,4, 100 mM NaCl, 0.1% Triton X-100) containing 0.025 µg/ml FLAG-peptide (Sigma), complexes were eluted by boiling in 40 µl 2 x SDS load buffer (4% SDS, 20% glycerol, 125 mM Tris-HCl, pH 6.8, 10% b-mercaptoethanol, 0.004% Bromphenol Blue).

### Western Blotting

Samples were mixed with 5x Loading buffer (Fermentas) and 20x Reducing agent (Fermentas) to a final concentration of 1x. The samples were heated to 95°C for 5 min and centrifuged for 1 min at 16000 rpm at room temperature. Samples were loaded onto a Tris-HCl Ready Gel 4–15% (Biorad) and ran at 70 V until the loading buffer had reached the bottom. Proteins were transferred to a Amersham hybond-P membrane (GE Healthcare) at 75 V for 30 min at 4°C, and the membrane was blocked in 10% skimmed milk powder (Difco) mixed with PBS and 1% Tween 20 (Sigma-Aldrich) over night at 4°C. The membrane was incubated with primary antibodies diluted in PTM buffer (PBS containing 0.5% skimmed milk powder and 0.1% Tween 20) for 1.5 h at 4°C. The primary antibodies used were mouse anti-TAF15 clone 8TA2B10 (a kind gift from Dr. Laszlo Tora), mouse anti-EWS (sc-48404 Santa Cruz), mouse anti-FUS (sc-47711 Santa Cruz), goat anti-TIA1 (sc-1751, C-20 Santa Cruz), goat anti-TIAR (sc-1749,C-18, Santa Cruz), all in 1/2000 dilutions, rabbit anti-FUS (Bethyl A300-302A Bethyl Laboratories), mouse anti-HuR (sc-5261, 3A2, Santa Cruz), and rabbit anti-CYP2E1 (ab-28146 Abcam) all in 1/5000 dilutions, and mouse anti-hnRNPC1/C2 in 1/10000 dilution. The membrane was washed in PTM buffer three times and incubated with secondary goat polyclonal HRP-conjugated antibodies (Dako) diluted 1∶10000 in PTM buffer 1 h at room temperature, and washed 5 times in PTM-buffer. Proteins were revealed using BM Chemo-luminescence Blotting substrate (POD) (Roche) and the signal was detected with X-ray film (Konica Minolta) developed with (AGFA Curix 60).

### Quantitative Real-Time PCR

RT-qPCR was performed on a Lightcycler 480 (Roche). All reactions were performed in triplicates in a total volume of 10 µl each using Lightcycler 480 SYBR Green I Master (Roche) according to manufacturer’s instructions. 1 µl cDNA was used as template and 3 pmol of each primer. Primer sequences are available upon request. Cycle conditions: 95°C 10 s, 58°C 20 s, 72°C 15 s, 40 repeats. The specificity of the reactions was confirmed by melting curve analysis and agarose gel electrophoresis. Agarose gel electrophoresis was performed by use of gels made of 1.2% agarose boiled with Tris-acetate-EDTA-buffer, and gels were run at 100 V. Primer efficiency was measured by dilution standard curves (above 95%). The amount of mRNA was normalized to the measured expression of GAPDH mRNA. The quantification was done as previously described [45].

### Whole Genome Expression Analysis

Gene expression profiling was conducted, using Illumina HumanGW 6 array, by AROS Applied Biotechnology, Aarhus, Denmark. Raw data were normalized with quantile normalization, followed by non-specific filtering using the computer program R (PMA call, SD removal) removing all probe sets that are not expressed in any array. Raw data is deposited in the NCBI’s Gene Expression Omnibus [47] and are accessible through GEO Series accession number GSE35578 (http://www.ncbi.nlm.nih.gov/geo/query/acc.cgi?acc=GSE35578. By this filtration, 22059 out of 48803 (45.2%) probe sets were removed. Background intensity was removed from the gene expression intensity. To determine the background intensity, the average intensity was summarized in the negative probe control in all arrays. The background intensity was determined to 28. The following criteria were used to determine the gene expression change in the siRNA transfection cells compared to control cells. Ratio  =  (siA_RI_-28)/(ctrlA_RI_-28), “siA_RI_” refers to the raw expression intensity of gene A in siRNA group and “ctrlA_RI_” refers to the raw expression intensity of gene A in control. As one array was used per treatment (n = 1) to determine the differentially expressed genes the following criteria were used. For higher expressed genes: Ratio >2 and detection p-value of gene expression in siRNA treatment group<0.05. For lower expressed genes: Ratio <0.5 and detection p-value of gene expression in control group<0.05. Hierarchical clustering was done with Pvclust [48], and pathway analysis was done with Ingenuity IPA 9.0 (Ingenuity Systems Inc.).

## Supporting Information

Figure S1 **Immunostainings of the FET-proteins and the TIA1 protein using monoclonal antibodies in SH-SY5Y cells after arsenite stress.** The nucleus is counterstained with DAPI. **A.** TIA1 and FUS immunostaining. The nuclei are counterstained by DAPI. **B.** TIA1 and TAF15 immunostaining. The nuclei are counterstained by DAPI **C.** TIA1 and EWS immunostaining. The nuclei are counterstained by DAPI.
(PDF)

Figure S2 **Immunostainings of the TIA1 protein in HEK293-cells after FUS+EWS+TAF15 knock-down and oxidative stress.** Cells were triple transfected with siRNAs against the FUS, EWS, and TAF15 mRNAs (siFET) or with an unspecific siRNA as control (siControl), and treated with arsenite to induce oxidative stress. **A.** TIA1 staining. The nucleus is counterstained with DAPI. **B.** The amounts of FUS, EWS, and TAF15 mRNA in the cells were measured by RT-qPCR and quantified against control siRNA treated cells. Standard deviations of technical triplicates are shown.
(PDF)

Figure S3 **FET protein expression in HEK293 and SH-SY5Y cells after oxidative stress.** Western blot showing FUS, EWS, and TAF15 protein expression in HEK293 and SH-SY5Y-cells treated with either H_2_O_2_ or arsenite to induce oxidative stress. Control cells are unstressed cells growing in the same type of growth media as the stressed cells (the H_2_O_2_ stress incubation is performed in media without fetal bovine serum). The expression of the hnRNPC1/C2 protein is shown as loading control. The expression of all three FET-proteins is unchanged by induction of oxidative stress by arsenite as well as H_2_O_2_ in both HEK293 and SH-SY5Y-cells.
(PDF)

Figure S4 **Effect of overexpression of the FET-proteins in HEK293 cells. A.** RT-qPCR analysis of the gene expression in FLAG-FUS, FLAG-EWS, or FLAG-TAF15 transfected HEK293 cells. RT-qPCR was performed in triplicates, and the gene expression was normalized to the expression of the housekeeping gene GAPDH, and quantified against the expression in cells transfected with an equal amount EGFP plasmid [45]. The expression level as measured by RT-qPCR is shown in black, and the measured level in the array is shown in white. Standard deviations from the technical triplicates are shown. **B.** FUS, EWS, and TAF15 protein expression in the FLAG-FUS, FLAG-EWS, and FLAG-TAF15 transfected HEK293 cells used in A. Control cells are transfected with an equal amount of EGFP plasmid. The expression of the hnRNPC1/C2 protein is shown as loading control.
(PDF)

Figure S5 **FET siRNA transfection of SH-SY5Y cells. A.** RT-qPCR analysis of the gene expression in SH-SY5Y cells transfected with siFUS, siEWS, and siTAF15. Candidate genes which expression is altered by siFUS, siEWS, and siTAF15 transfection in HEK293 cells are chosen. The expression level as measured by RT-qPCR in transfected SH-SY5Y cells is shown in black, and the level in HEK293 transfected cells by the expression array is shown in white. RT-qPCR was performed in triplicates, and the gene expression was normalized to the expression of the housekeeping gene GAPDH and quantified [45]. Standard deviations from the technical triplicates are shown. **B.** FUS, EWS and TAF15 protein expression in siFUS, siEWS, and siTAF15 transfected SH-SY5Y cells used in A. Control cells are transfected with an equal amount of unspecific siRNA. The protein expression of hnRNPC1/C2 is shown as loading control.
(PDF)

Figure S6 **Western Blot of HEK293-cells to detect CYP2E1-expression. A.** HEK293-cells were transfected with siRNAs targeted against the FUS, EWS, and TAF15 mRNAs, or with an unspecific siRNA as control. Protein extracted from pig liver is used as positive control of CYP2E1 protein expression. siFET denotes that cells were transfected simultaneously with siFUS+siEWS+siTAF15, siControl+++ denotes control cells were transfected with an equal amount of unspecific siRNA. Lanes 1–7 are blotted with a polyclonal antibody against the CYP2E1 protein (50–55 kDa). CYP2E1-expression is only detected in the positive control. **B.** HEK293-cells were simultaneously transfected with siFUS+siEWS+siTAF15 (siFET), or with an equal amount of unspecific siRNA (siControl+++) as control. To induce expression of the CYP2E1-protein 48 h after the transfection cells were treated with 100 mM ethanol for 18 h. Protein extracted from pig liver is used as positive control of CYP2E1 protein expression. Lanes 1–5 are blotted with a polyclonal antibody against the CYP2E1 protein (50–55 kDa), and lanes 6–9 with an antibody against the hnRNPC1/C2 protein (41+43 kDa) as a loading control. In lanes 6–9 it is loaded 1/8 of the volume loaded in lanes 2–5. CYP2E1-expression is only detected in the positive control. **C.** HEK293-cells were simultaneously transfected with siFUS+siEWS+siTAF15 (siFET), or with an equal amount of unspecific siRNA (siControl+++) as control. To induce the expression of CYP2E1-protein 48 h after the transfection cells were treated with DMSO for 24 h. Protein extracted from pig liver is used as positive control of CYP2E1 protein expression. Lanes 1–5 are blotted with a polyclonal antibody against the CYP2E1 protein (50–55 kDa). CYP2E1-expression is only detected in the positive control.
(PDF)

Figure S7 **FET-protein functions.** Pathway analysis by Ingenuity IPA 9.0 of the DEGs in siFUS, siEWS, siTAF15, and siFUS+EWS+TAF15 transfected cells. Five of the categories identified in each sample by the Bio Functions analysis are shown. The –log(p-value) is shown on the y-axis, and the threshold line (p = 0.05) is shown in yellow.
(PDF)

Table S1 **Stress granule formation after FUS, EWS, and TAF15 knock-down.** Number of stress granules after FET siRNA knock-down and arsenite stress, compared to control siRNA. Cells were photographed at five different locations and SGs were counted using the ImageJ software. The counted SGs in each frame were then divided by the total number of cells in that frame to calculate an average SG content per cell and standard deviation.
(DOCX)

Table S2 **Quantification of ROS-content.** Pictures taken after the oxidative stress assay were analyzed by the ImageJ software and the mean pixel intensities were measured. Standard deviations are calculated from three pictures of each treatment.
(DOCX)

Table S3 **List of commonly identified DEGs.** The genes are also displayed by visual graphics in Figure 6B.
(DOCX)

Table S4 **The 10 most up regulated genes in FET siRNA transfected HEK293 cells.**
(DOCX)

Table S5 **The 10 most down regulated genes in FET siRNA transfected HEK293 cells.**
(DOCX)

Table S6 **List of DEGs.** DEGs in HEK293-cells after siRNA mediated gene knock-down of the FUS, EWS, TAF15, or FUS+EWS+TAF15 proteins.
(XLSX)

Table S7 **IPA Bio Functions.** Pathway analysis by IPA of DEGs in HEK293-cells after siRNA mediated gene knock-down of the FUS, EWS, TAF15, or FUS+EWS+TAF15 proteins. Five categories of the Bio Functions analysis belonging to each siRNA group are listed.
(DOCX)

Table S8 **Total list of IPA Bio Functions.** Pathway analysis by IPA of DEGs in HEK293-cells after siRNA mediated gene knock-down of the FUS, EWS, TAF15, or FUS+EWS+TAF15 proteins. The total categories of the Bio Functions analysis belonging to each siRNA group are listed.
(XLSX)

Table S9 **IPA category neurological disease.** Pathway analysis by IPA of DEGs in HEK293-cells after siRNA mediated gene knock-down of the FUS, EWS, TAF15, or FUS+EWS+TAF15 proteins. The category Neurological Disease of the Bio Functions analysis belonging to each siRNA group is listed.
(XLSX)

Table S10 **IPA toxicity lists.** Pathway analysis by IPA of DEGs in HEK293-cells after siRNA mediated gene knock-down of the FUS, EWS, TAF15, or FUS+EWS+TAF15 proteins. The Ingenuity Toxicity Lists belonging to each siRNA group are listed.
(XLSX)

Table S11 **Comparison of DEGs and genes identified by CLIP by Hoell et al., 2011.**
(XLSX)
