# Supplementary figures and images for: Gene Expression Responses to FUS, EWS, and TAF15 Reduction and Stress Granule Sequestration Analyses Identifies FET-Protein Non-Redundant Functions
Source: PLoS One. 2012 Sep 25;7(9):e46251. doi: 10.1371/journal.pone.0046251 (PMC3457980; doi:10.1371/journal.pone.0046251)

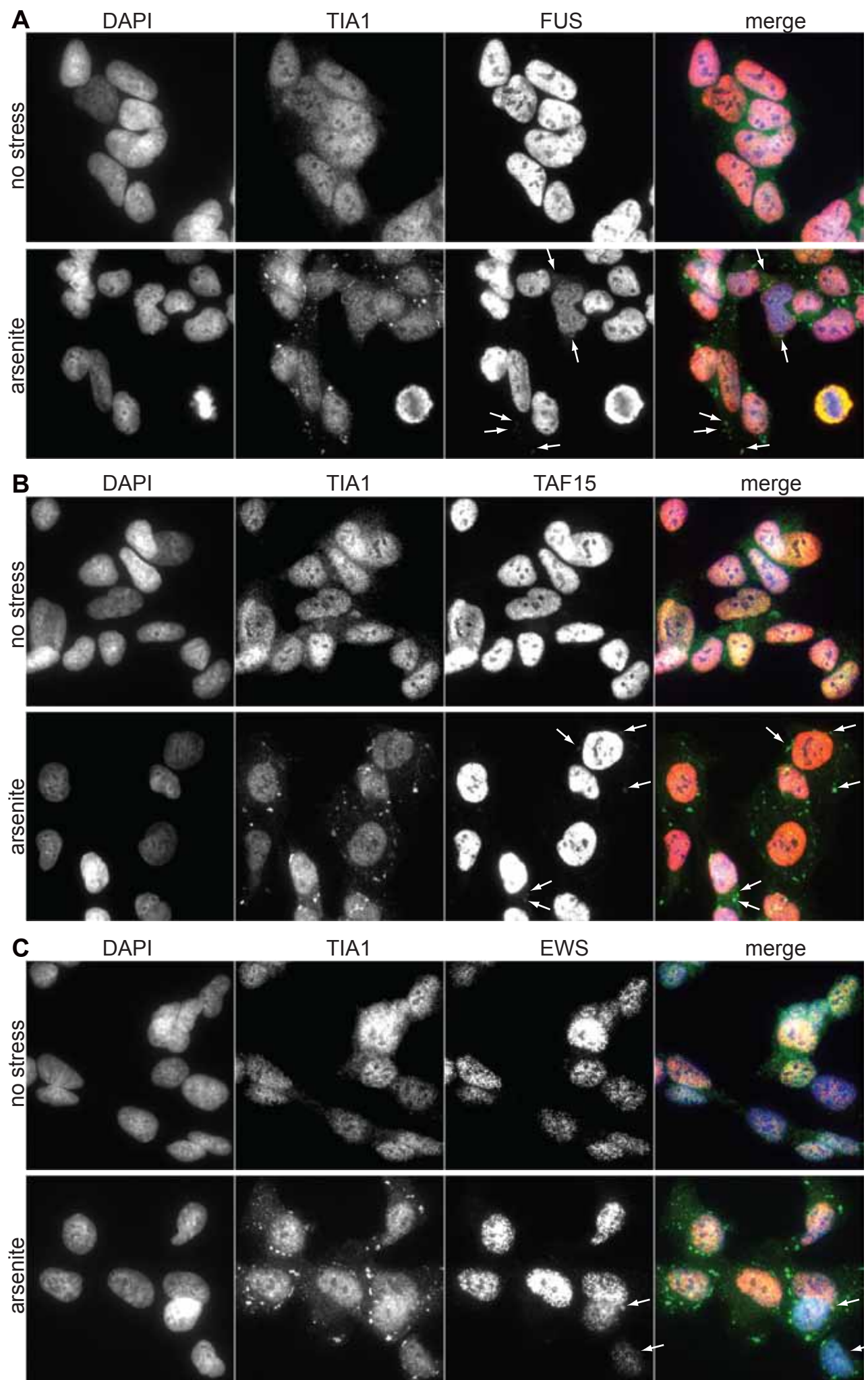

Figure S1

Supplement: Figure S1 — Immunostainings of the FET-proteins and the TIA1 protein using monoclonal antibodies in SH-SY5Y cells after arsenite stress. The nucleus is counterstained with DAPI. A. TIA1 and FUS immunostaining. The nuclei are counterstained by DAPI. B. TIA1 and TAF15 immunostaining. The nuclei are counterstained by DAPI C. TIA1 and EWS immunostaining. The nuclei are counterstained by DAPI. (PDF) [file pone.0046251.s001.pdf]

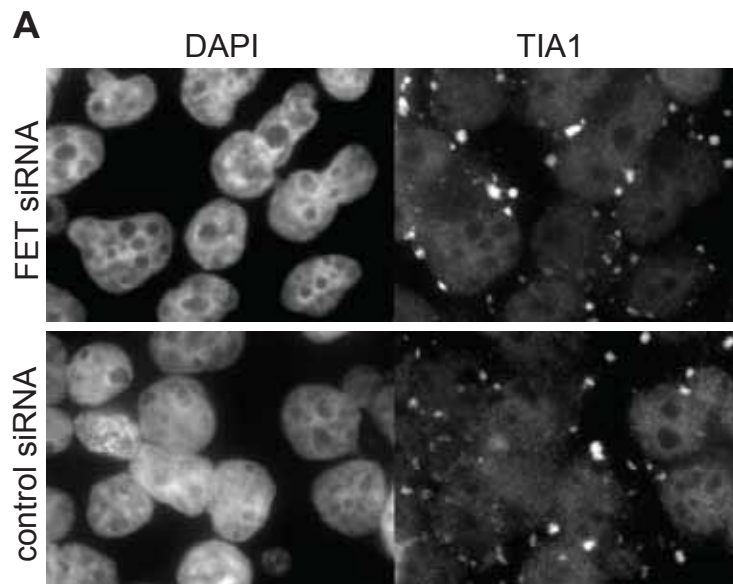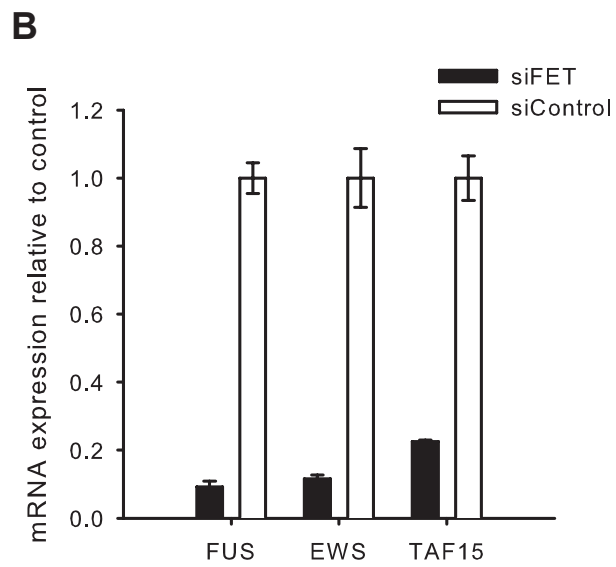

Figure S2

Supplement: Figure S2 — Immunostainings of the TIA1 protein in HEK293-cells after FUS+EWS+TAF15 knock-down and oxidative stress. Cells were triple transfected with siRNAs against the FUS, EWS, and TAF15 mRNAs (siFET) or with an unspecific siRNA as control (siControl), and treated with arsenite to induce oxidative stress. A. TIA1 staining. The nucleus is counterstained with DAPI. B. The amounts of FUS, EWS, and TAF15 mRNA in the cells were measured by RT-qPCR and quantified against control siRNA treated cells. Standard deviations of technical triplicates are shown. (PDF) [file pone.0046251.s002.pdf]

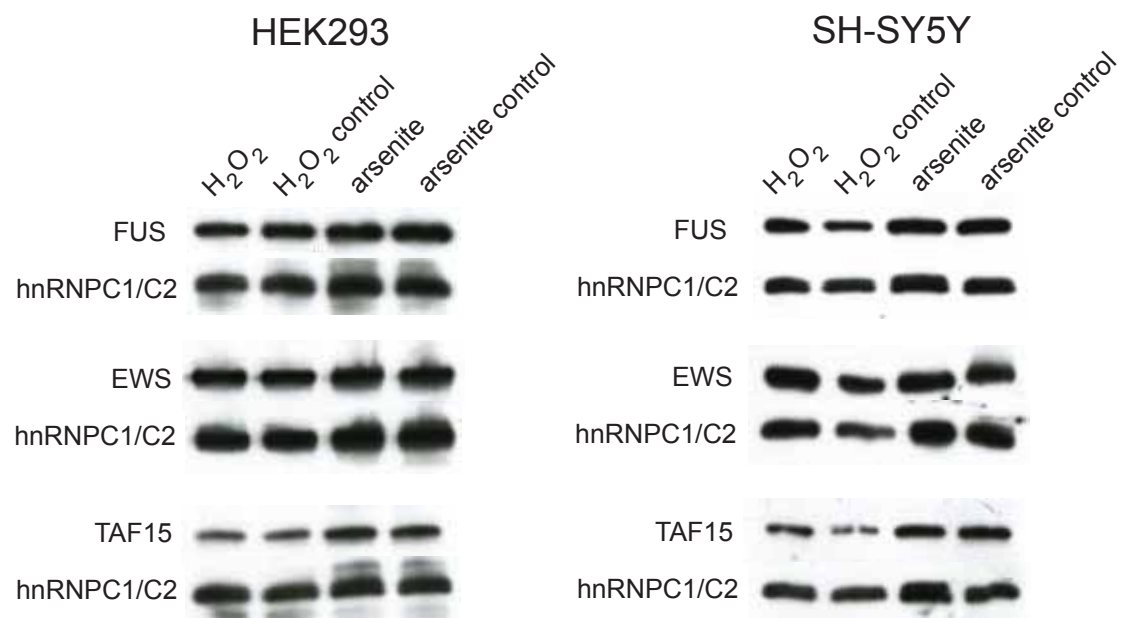

Figure S3

Supplement: Figure S3 — FET protein expression in HEK293 and SH-SY5Y cells after oxidative stress. Western blot showing FUS, EWS, and TAF15 protein expression in HEK293 and SH-SY5Y-cells treated with either H2O2 or arsenite to induce oxidative stress. Control cells are unstressed cells growing in the same type of growth media as the stressed cells (the H2O2 stress incubation is performed in media without fetal bovine serum). The expression of the hnRNPC1/C2 protein is shown as loading control. The expression of all three FET-proteins is unchanged by induction of oxidative stress by arsenite as well as H2O2 in both HEK293 and SH-SY5Y-cells. (PDF) [file pone.0046251.s003.pdf]

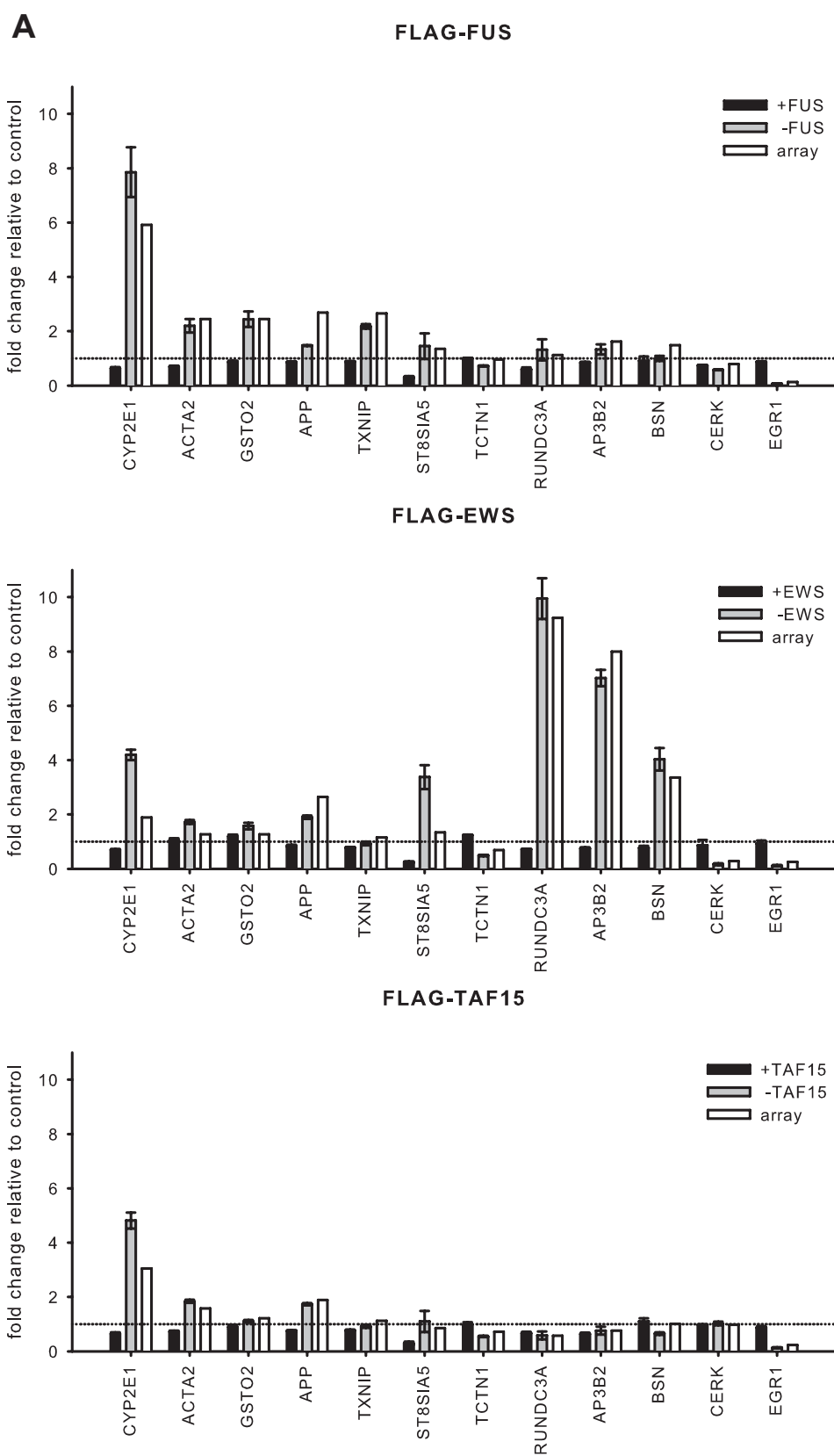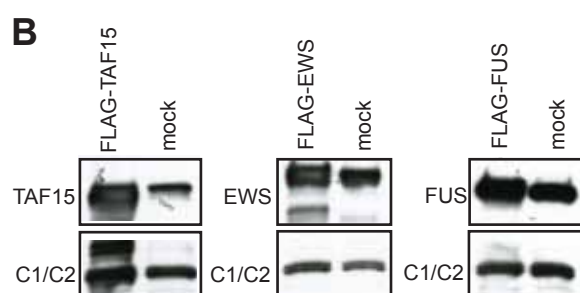

Figure S4

Supplement: Figure S4 — Effect of overexpression of the FET-proteins in HEK293 cells. A. RT-qPCR analysis of the gene expression in FLAG-FUS, FLAG-EWS, or FLAG-TAF15 transfected HEK293 cells. RT-qPCR was performed in triplicates, and the gene expression was normalized to the expression of the housekeeping gene GAPDH, and quantified against the expression in cells transfected with an equal amount EGFP plasmid [45]. The expression level as measured by RT-qPCR is shown in black, and the measured level in the array is shown in white. Standard deviations from the technical triplicates are shown. B. FUS, EWS, and TAF15 protein expression in the FLAG-FUS, FLAG-EWS, and FLAG-TAF15 transfected HEK293 cells used in A. Control cells are transfected with an equal amount of EGFP plasmid. The expression of the hnRNPC1/C2 protein is shown as loading control. (PDF) [file pone.0046251.s004.pdf]

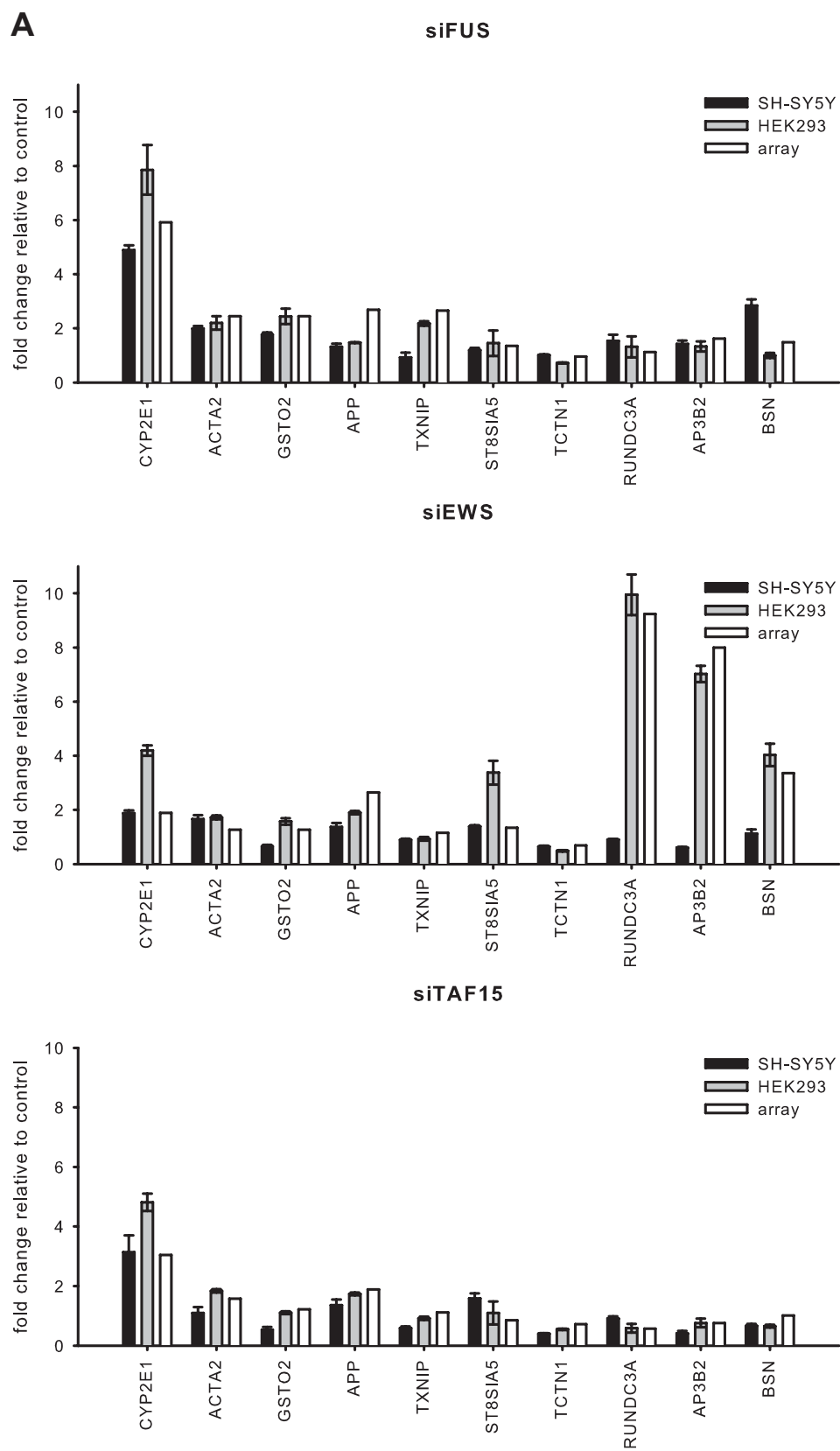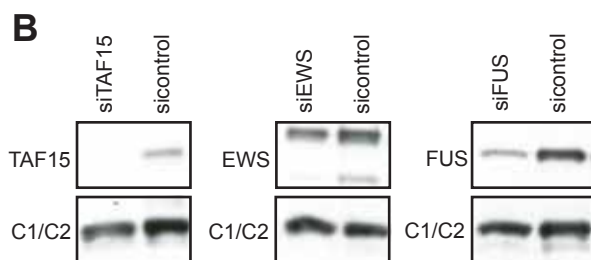

Figure S5

Supplement: Figure S5 — FET siRNA transfection of SH-SY5Y cells. A. RT-qPCR analysis of the gene expression in SH-SY5Y cells transfected with siFUS, siEWS, and siTAF15. Candidate genes which expression is altered by siFUS, siEWS, and siTAF15 transfection in HEK293 cells are chosen. The expression level as measured by RT-qPCR in transfected SH-SY5Y cells is shown in black, and the level in HEK293 transfected cells by the expression array is shown in white. RT-qPCR was performed in triplicates, and the gene expression was normalized to the expression of the housekeeping gene GAPDH and quantified [45]. Standard deviations from the technical triplicates are shown. B. FUS, EWS and TAF15 protein expression in siFUS, siEWS, and siTAF15 transfected SH-SY5Y cells used in A. Control cells are transfected with an equal amount of unspecific siRNA. The protein expression of hnRNPC1/C2 is shown as loading control. (PDF) [file pone.0046251.s005.pdf]

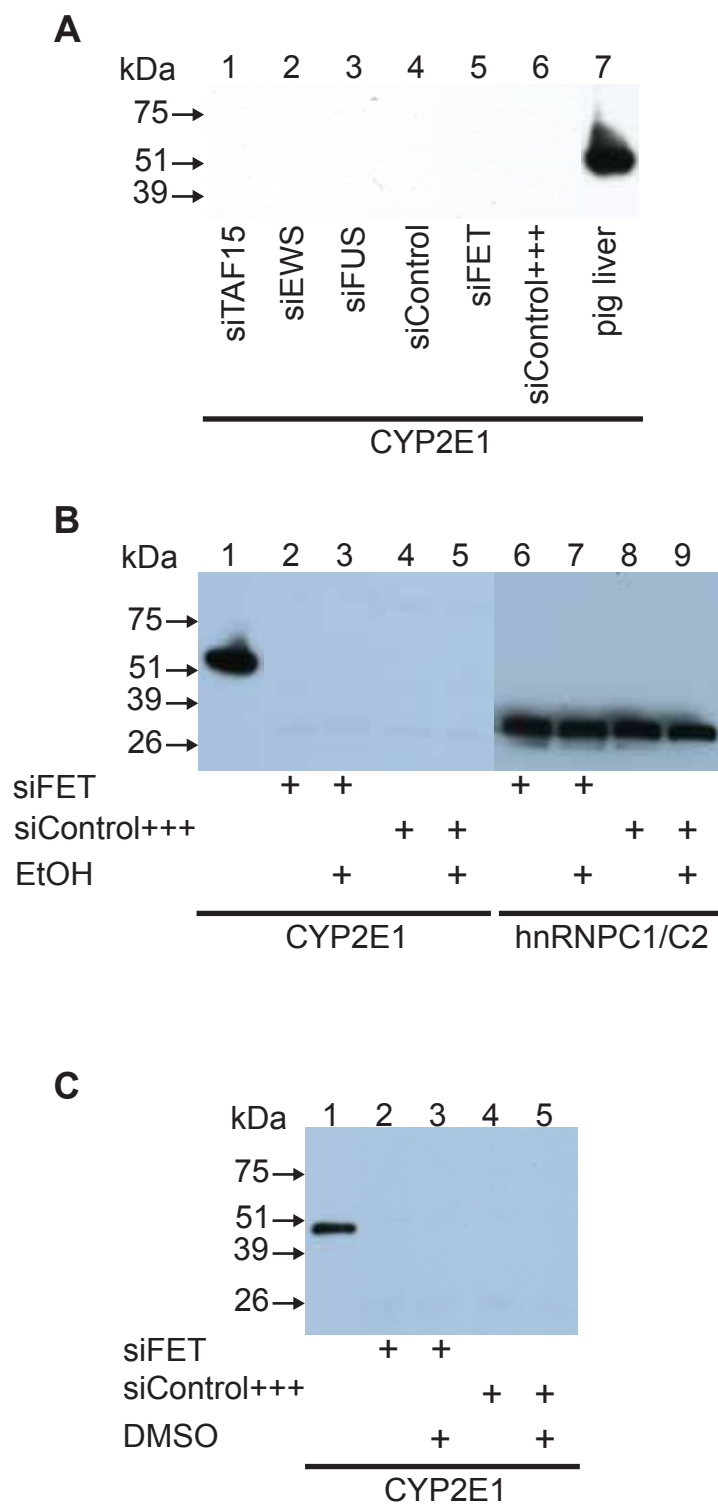

Figure S6

Supplement: Figure S6 — Western Blot of HEK293-cells to detect CYP2E1-expression. A. HEK293-cells were transfected with siRNAs targeted against the FUS, EWS, and TAF15 mRNAs, or with an unspecific siRNA as control. Protein extracted from pig liver is used as positive control of CYP2E1 protein expression. siFET denotes that cells were transfected simultaneously with siFUS+siEWS+siTAF15, siControl+++ denotes control cells were transfected with an equal amount of unspecific siRNA. Lanes 1–7 are blotted with a polyclonal antibody against the CYP2E1 protein (50–55 kDa). CYP2E1-expression is only detected in the positive control. B. HEK293-cells were simultaneously transfected with siFUS+siEWS+siTAF15 (siFET), or with an equal amount of unspecific siRNA (siControl+++) as control. To induce expression of the CYP2E1-protein 48 h after the transfection cells were treated with 100 mM ethanol for 18 h. Protein extracted from pig liver is used as positive control of CYP2E1 protein expression. Lanes 1–5 are blotted with a polyclonal antibody against the CYP2E1 protein (50–55 kDa), and lanes 6–9 with an antibody against the hnRNPC1/C2 protein (41+43 kDa) as a loading control. In lanes 6–9 it is loaded 1/8 of the volume loaded in lanes 2–5. CYP2E1-expression is only detected in the positive control. C. HEK293-cells were simultaneously transfected with siFUS+siEWS+siTAF15 (siFET), or with an equal amount of unspecific siRNA (siControl+++) as control. To induce the expression of CYP2E1-protein 48 h after the transfection cells were treated with DMSO for 24 h. Protein extracted from pig liver is used as positive control of CYP2E1 protein expression. Lanes 1–5 are blotted with a polyclonal antibody against the CYP2E1 protein (50–55 kDa). CYP2E1-expression is only detected in the positive control. (PDF) [file pone.0046251.s006.pdf]

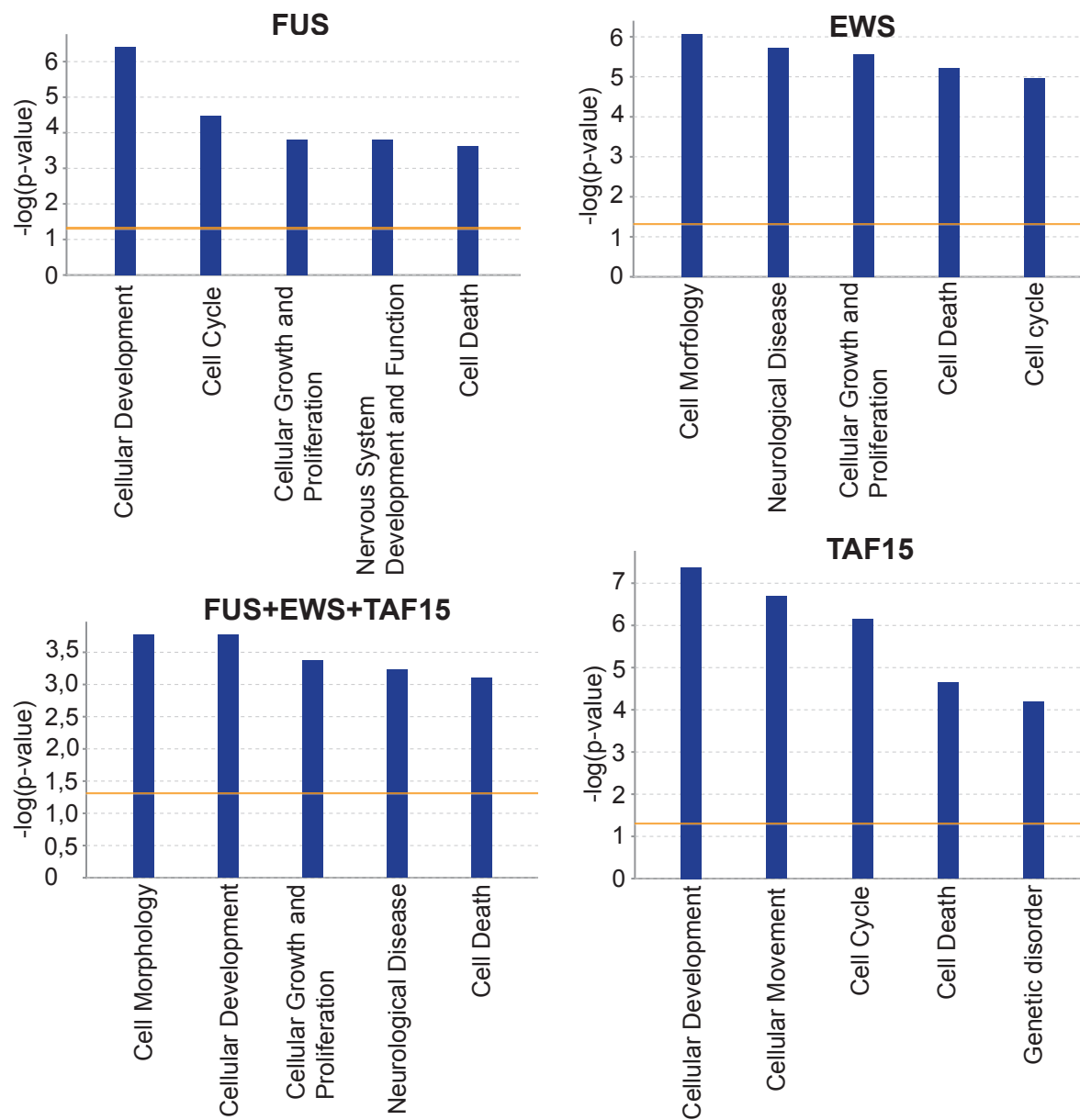

**Figure S7**

Supplement: Figure S7 — FET-protein functions. Pathway analysis by Ingenuity IPA 9.0 of the DEGs in siFUS, siEWS, siTAF15, and siFUS+EWS+TAF15 transfected cells. Five of the categories identified in each sample by the Bio Functions analysis are shown. The –log(p-value) is shown on the y-axis, and the threshold line (p = 0.05) is shown in yellow. (PDF) [file pone.0046251.s007.pdf]
