# Supplementary material for: Gene Expression Responses to FUS, EWS, and TAF15 Reduction and Stress Granule Sequestration Analyses Identifies FET-Protein Non-Redundant Functions
Source: PLoS One. 2012 Sep 25;7(9):e46251. doi: 10.1371/journal.pone.0046251 (PMC3457980; doi:10.1371/journal.pone.0046251)
Supplement: Table S1 — Stress granule formation after FUS, EWS, and TAF15 knock-down. Number of stress granules after FET siRNA knock-down and arsenite stress, compared to control siRNA. Cells were photographed at five different locations and SGs were counted using the ImageJ software. The counted SGs in each frame were then divided by the total number of cells in that frame to calculate an average SG content per cell and standard deviation. (DOCX) [file pone.0046251.s008.docx]

| **Supplementary Table S1. Number of stress granules after FET siRNA knock-down and arsenite stress, compared to control siRNA.** | | | | | | |
| --- | --- | --- | --- | --- | --- | --- |
| **antibody** | **siRNA** | ***N* SGs** | ***N* cells** | **SG/cell** | **mean** | **stdv** |
| **FUS + TIA1** | **FUS** | 106 | 50 | 2.120 | 2.265 | 0.206 |
|  |  | 125 | 54 | 2.315 |  |  |
|  |  | 105 | 54 | 1.944 |  |  |
|  |  | 127 | 51 | 2.490 |  |  |
|  |  | 81 | 33 | 2.455 |  |  |
|  | **Control** | 58 | 25 | 2.320 | 2.548 | 0.499 |
|  |  | 85 | 29 | 2.931 |  |  |
|  |  | 116 | 36 | 3.222 |  |  |
|  |  | 57 | 32 | 1.781 |  |  |
|  |  | 82 | 33 | 2.485 |  |  |
| **EWS + TIA1** | **EWS** | 128 | 61 | 2.098 | 2.349 | 0.262 |
|  |  | 122 | 49 | 2.490 |  |  |
|  |  | 102 | 50 | 2.040 |  |  |
|  |  | 111 | 47 | 2.362 |  |  |
|  |  | 135 | 49 | 2.755 |  |  |
|  | **Control** | 108 | 48 | 2.250 | 2.460 | 0.414 |
|  |  | 137 | 66 | 2.076 |  |  |
|  |  | 117 | 55 | 2.127 |  |  |
|  |  | 146 | 46 | 3.174 |  |  |
|  |  | 123 | 46 | 2.674 |  |  |
| **TAF15 + TIA1** | **TAF15** | 115 | 49 | 2.347 | 2.304 | 0.503 |
|  |  | 53 | 20 | 2.650 |  |  |
|  |  | 44 | 19 | 2.316 |  |  |
|  |  | 22 | 16 | 1.375 |  |  |
|  |  | 85 | 30 | 2.833 |  |  |
|  | **Control** | 42 | 16 | 2.625 | 2.410 | 0.184 |
|  |  | 54 | 21 | 2.571 |  |  |
|  |  | 57 | 26 | 2.192 |  |  |
|  |  | 84 | 31 | 2.710 |  |  |
|  |  | 72 | 30 | 2.400 |  |  |
| **TIA1** | **FUS + EWS + TAF15** | 50 | 40 | 1.25 | 2.517 | 0.835 |
|  |  | 113 | 48 | 2.35 |  |  |
|  |  | 168 | 70 | 2.40 |  |  |
|  |  | 158 | 58 | 2.72 |  |  |
|  |  | 135 | 35 | 3.86 |  |  |
|  | **Control** | 90 | 35 | 2.57 | 2.491 | 0.603 |
|  |  | 75 | 24 | 3.13 |  |  |
|  |  | 67 | 49 | 1.37 |  |  |
|  |  | 115 | 40 | 2.88 |  |  |
|  |  | 78 | 31 | 2.52 |  |  |
