# Supplementary material for: Gene Expression Responses to FUS, EWS, and TAF15 Reduction and Stress Granule Sequestration Analyses Identifies FET-Protein Non-Redundant Functions
Source: PLoS One. 2012 Sep 25;7(9):e46251. doi: 10.1371/journal.pone.0046251 (PMC3457980; doi:10.1371/journal.pone.0046251)
Supplement: Table S2 — Quantification of ROS-content. Pictures taken after the oxidative stress assay were analyzed by the ImageJ software and the mean pixel intensities were measured. Standard deviations are calculated from three pictures of each treatment. (DOCX) [file pone.0046251.s009.docx]

| **Supplementary Table S2. Quantification of ROS-content.** | | | |
| --- | --- | --- | --- |
| **Figure** | **Treatment** | **Mean Pixel Intensity** | **±SD** |
| **5A** | no siRNA +TBHP | 46.36 | 3.922 |
|  | no siRNA -TBHP | 21.21 | 0.009 |
|  | siFUS | 23.16 | 0.042 |
|  | siEWS | 16.86 | 2.610 |
|  | siTAF15 | 21.35 | 0.968 |
|  | si control 100 nM | 21.22 | 0.664 |
|  | siFUS+EWS+TAF15 | 32.56 | 1.354 |
|  | si control 300 nM | 26.92 | 0.288 |
| **5B** | no siRNA +TBHP | 30.59 | 1.059 |
|  | no siRNA -TBHP | 17.83 | 1.149 |
|  | FUS+EWS+TAF15 siRNA +TBHP | 27.71 | 3.740 |
|  | FUS+EWS+TAF15 siRNA -TBHP | 19.44 | 2.373 |
|  | sicontrol 300 nM +TBHP | 23.31 | 1.852 |
|  | sicontrol 300 nM -TBHP | 16.47 | 1.946 |
