# Supplementary material for: Gene Expression Responses to FUS, EWS, and TAF15 Reduction and Stress Granule Sequestration Analyses Identifies FET-Protein Non-Redundant Functions
Source: PLoS One. 2012 Sep 25;7(9):e46251. doi: 10.1371/journal.pone.0046251 (PMC3457980; doi:10.1371/journal.pone.0046251)
Supplement: Table S3 — List of commonly identified DEGs. The genes are also displayed by visual graphics in Figure 6B. (DOCX) [file pone.0046251.s010.docx]

| **Supplementary Table S3. Commonly identified DEGs after FUS, EWS, or TAF15 knock-down** | | | | | | | |
| --- | --- | --- | --- | --- | --- | --- | --- |
| **FUS and EWS** | | **FUS and TAF15** | | **EWS and TAF15** | | **FUS, EWS, and TAF15** | |
| **PROBE_ID** | **SYMBOL** | **PROBE_ID** | **SYMBOL** | **PROBE_ID** | **SYMBOL** | **PROBE_ID** | **SYMBOL** |
| ILMN_2139970 | ALDH1A3 | ILMN_1686846 | AKAP12 | ILMN_1759261 | C16orf55 | ILMN_1728742 | C5orf4 |
| ILMN_1653283 | APP | ILMN_1684694 | ANK1 | ILMN_1801703 | CPLX1 | ILMN_1806951 | CSTF3 |
| ILMN_1729288 | C1QTNF6 | ILMN_2322747 | ARHGAP5 | ILMN_1762002 | CSTF3 | ILMN_1777164 | DIP2A |
| ILMN_2149226 | CAV1 | ILMN_1665437 | CYP2E1 | ILMN_1651752 | CXorf21 | ILMN_1842426 |  |
| ILMN_1753468 | CD63 | ILMN_1878375 |  | ILMN_2122300 | DNAL1 | ILMN_1835466 |  |
| ILMN_1773079 | COL3A1 | ILMN_2163070 | KHDC1 | ILMN_1805404 | GRIN1 | ILMN_1801090 | KRT222P |
| ILMN_1775931 | EPHA3 | ILMN_2152422 | MCART6 | ILMN_1898136 |  | ILMN_1693685 | LOC205251 |
| ILMN_1754921 | FAM43B | ILMN_1722718 | BMP2 | ILMN_1891482 |  | ILMN_2156936 | SCCPDH |
| ILMN_1690532 | FLJ40473 | ILMN_1742473 | C10orf72 | ILMN_1765668 | IL20RB | ILMN_1762899 | EGR1 |
| ILMN_1729905 | GAL3ST1 | ILMN_1797009 | F3 | ILMN_1796751 | KIAA1274 | ILMN_1753830 | ETV4 |
| ILMN_1807925 | GNG2 | ILMN_1654566 | HSPA1L | ILMN_1749304 | LOC643389 | ILMN_1739222 | ETV5 |
| ILMN_1654319 | HAPLN3 | ILMN_1653276 | LOC645276 | ILMN_1746343 | LOC643985 | ILMN_1669523 | FOS |
| ILMN_1881081 |  | ILMN_1751016 | LONRF2 | ILMN_1740707 | LOC646123 | ILMN_1751607 | FOSB |
| ILMN_1721758 | ID4 | ILMN_1680139 | MAFF | ILMN_1803419 | LOC652610 | ILMN_1677092 | GEM |
| ILMN_1810628 | KIAA0367 | ILMN_1712523 | MAP6 | ILMN_2190676 | LRTM2 | ILMN_1910712 |  |
| ILMN_2183424 | LOC374491 | ILMN_1675927 | NEBL | ILMN_2050183 | MYLK2 | ILMN_1740181 | LOC283345 |
| ILMN_1672656 | LOC645733 | ILMN_2330310 | OR4L1 | ILMN_1653390 | SDHALP1 | ILMN_1689057 | PIK3R1 |
| ILMN_2227162 | LOC653305 | ILMN_1735745 | OSTN | ILMN_1788251 | SNN | ILMN_1784822 | PPP1R3F |
| ILMN_2343097 | NCALD | ILMN_1733045 | RAB36 | ILMN_1701637 | SULT4A1 | ILMN_1760412 | SHISA2 |
| ILMN_1670535 | NDRG2 | ILMN_1675979 | RBMS3 | ILMN_2196734 | HRB | ILMN_2086105 | SPRY4 |
| ILMN_1685750 | RAB3C | ILMN_2252701 | SLC6A9 | ILMN_1840923 |  | ILMN_2074894 | TREML2P |
| ILMN_2376502 | RHOBTB1 | ILMN_2093188 | USP43 | ILMN_1847231 |  |  |  |
| ILMN_2096623 | SLITRK1 |  |  | ILMN_1697907 | LOC649542 |  |  |
| ILMN_1704500 | STAP2 |  |  | ILMN_1743619 | NEDD9 |  |  |
| ILMN_1763333 | TBX6 |  |  | ILMN_2054297 | PTGS2 |  |  |
| ILMN_1706969 | C6orf65 |  |  | ILMN_1655229 | SLC7A11 |  |  |
| ILMN_1739576 | CYB5R2 |  |  | ILMN_1719759 | TNC |  |  |
| ILMN_2341661 | ETV4 |  |  | ILMN_1803882 | VEGFA |  |  |
| ILMN_2367883 | GEM |  |  | ILMN_1679984 | ZCCHC12 |  |  |
| ILMN_2115340 | HIST2H4A |  |  |  |  |  |  |
| ILMN_2086077 | JUNB |  |  |  |  |  |  |
| ILMN_1749546 | LOC440013 |  |  |  |  |  |  |
| ILMN_1700763 | LOC643712 |  |  |  |  |  |  |
| ILMN_1784094 | PRDM16 |  |  |  |  |  |  |
| ILMN_1712112 | RCAN1 |  |  |  |  |  |  |
| ILMN_1791232 | SPRED2 |  |  |  |  |  |  |
| ILMN_1713161 | USP16 |  |  |  |  |  |  |
| ILMN_1804945 | ZNF667 |  |  |  |  |  |  |
