# Supplementary material for: Gene Expression Responses to FUS, EWS, and TAF15 Reduction and Stress Granule Sequestration Analyses Identifies FET-Protein Non-Redundant Functions
Source: PLoS One. 2012 Sep 25;7(9):e46251. doi: 10.1371/journal.pone.0046251 (PMC3457980; doi:10.1371/journal.pone.0046251)
Supplement: Table S4 — The 10 most up regulated genes in FET siRNA transfected HEK293 cells. (DOCX) [file pone.0046251.s011.docx]

| **Table S4.** The 10 most up regulated genes in FET siRNA transfected HEK293 cells | | | | |
| --- | --- | --- | --- | --- |
| **siRNA** | **GeneSymbol** | **Accession number** | **Function** | **Ratio** |
| **FUS** | *SLITRK1* | NM_052910.1 | neurite outgrowth | 7.31 |
|  | *CYP2E1* | NM_000773.3 | drug metabolism, synthesis of lipids | 5.91 |
|  | *SMURF1* | NM_020429.1 | ubiquitin ligase | 5.83 |
|  | *NEUROD6* | NM_022728.2 | transcription factor | 3.67 |
|  | *ADAM10* | NM_001110.2 | cleavage of proteins | 3.45 |
|  | *KRT222P* | NM_152349.2 | unknown | 3.44 |
|  | *COL3A1* | NM_000090.3 | pro-alpha1 chain of type III collagen | 3.36 |
|  | *GPR26* | NM_153442.1 | G protein-coupled receptor | 3.34 |
|  | *DIP2A* | NM_206891.1 | axon patterning of the CNS? | 3.34 |
|  | *NDRG2* | NM_201539.1 | neurite outgrowth | 3.23 |
| **EWS** | *SLITRK1* | NM_052910.1 | neurite outgrowth | 11.5 |
|  | *RUNDC3A* | NM_006695.3 | effector of RAP2A in neurons? | 9.24 |
|  | *AP3B2* | NM_004644.3 | neurotransmitter release | 8.00 |
|  | *ACTL6B* | NM_016188.3 | chromatin remodeling in brain | 4.88 |
|  | *CPLX1* | NM_006651.3 | neurotransmitter release | 4.74 |
|  | *RAD51L1* | NM_002877.4 | DNA damage | 4.66 |
|  | *C10orf132* | NM_001010917.1 | unknown | 4.59 |
|  | *SYP* | NM_003179.2 | protein in synaptic vesicles | 4.57 |
|  | *TNRC4* | NM_007185.3 | pre-mRNA alternative splicing | 4.24 |
|  | *CHGA* | NM_001275.3 | in secretory vesicles of neurons | 4.12 |
| **TAF15** | *N.A.* | BF879908 | unknown | 4.37 |
|  | *TMBIM1* | NM_022152.4 | unknown | 4.13 |
|  | *N.A.* | BF239137 | unknown | 4.09 |
|  | *TMEM19* | NM_018279.3 | unknown | 3.50 |
|  | *LOC440104* | XM_927712.2 | pseudogene | 3.32 |
|  | *CYP2E1* | NM_000773.3 | drug metabolism, synthesis of lipids | 3.05 |
|  | *CYB561* | NM_001017918.1 | electron transport secretory vesicle | 3.04 |
|  | *LOC643985* | XM_927235.1 | unknown | 2.96 |
|  | *GCET2* | NM_001008756.1 | signal transduction | 2.93 |
|  | *LOC126147* | NM_145807.1 | unknown | 2.85 |
| **FUS+EWS+TAF15** | *CYP2E1* | NM_000773.3 | drug metabolism, synthesis of lipids | 17.2 |
|  | *FOXK1* | NM_001037165.1 | regulator of myoglobin gene exp. | 6.56 |
|  | *N.A.* | AA534202 | unknown | 6.20 |
|  | *ST8SIA5* | NM_013305.4 | synthesis of gangliosides | 5.79 |
|  | *N.A.* | BX115156 | unknown | 4.96 |
|  | *DDX12* | XM_931895.1 | DNA helicase, cellular proliferation | 4.51 |
|  | *BAGE3* | NM_182481.1 | unknown | 4.46 |
|  | *ABCF2* | NM_005692.3 | ABC-transporter, cancer | 4.30 |
|  | *N.A.* | AI887987 | unknown | 4.27 |
|  | *DCDC5* | NM_198462.2 | microtubule polymerization | 4.23 |
