# Supplementary material for: Gene Expression Responses to FUS, EWS, and TAF15 Reduction and Stress Granule Sequestration Analyses Identifies FET-Protein Non-Redundant Functions
Source: PLoS One. 2012 Sep 25;7(9):e46251. doi: 10.1371/journal.pone.0046251 (PMC3457980; doi:10.1371/journal.pone.0046251)
Supplement: Table S5 — The 10 most down regulated genes in FET siRNA transfected HEK293 cells. (DOCX) [file pone.0046251.s012.docx]

| **Table S5.** The 10 most down regulated genes in FET siRNA transfected HEK293 cells | | | | |
| --- | --- | --- | --- | --- |
| **siRNA** | **GeneSymbol** | **Accession number** | **Function** | **Ratio** |
| **FUS** | *ETV5* | NM_004454.1 | transcription factor | 0.09 |
|  | *EGR1* | NM_001964.2 | transcriptional regulator | 0.13 |
|  | *FOS* | NM_005252.2 | cell proliferation, differentiation | 0.15 |
|  | *RCAN1* | NM_203418.1 | CNS development, Down syndrome | 0.18 |
|  | *N.A.* | CR738291 | unknown | 0.23 |
|  | *C19orf23* | NM_152480.1 | unknown | 0.24 |
|  | *AGPAT4* | NM_001012733.1 | phospholipid biosynthesis | 0.25 |
|  | *GEM* | NM_181702.1 | GTP-binding protein | 0.25 |
|  | *PLXND1* | NM_015103.1 | cell migration, proliferation, survival | 0.26 |
|  | *MAP6* | NM_207577.1 | microtubule stabilization | 0.28 |
| **EWS** | *ETV5* | NM_004454.1 | transcription factor | 0.14 |
|  | *RCAN1* | NM_203418.1 | CNS development, Down syndrome | 0.22 |
|  | *LOC644584* | XR_019152.1 | EWS pseudogene | 0.23 |
|  | *LOC389286* | NM_001018022.1 | repetitive element | 0.23 |
|  | *DUSP4* | NM_001394.5 | inactivates ERK1, ERK2 and JNK | 0.24 |
|  | *EGR1* | NM_001964.2 | transcriptional regulator | 0.26 |
|  | *INHBE* | NM_031479.3 | pancreatic exocrine cell growth | 0.26 |
|  | *CERK* | NM_182661.1 | proliferation, apoptosis | 0.26 |
|  | *SLC7A11* | NM_014331.3 | cystine and glutamate transport | 0.27 |
|  | *SPRY4* | NM_030964.2 | inhibitor of MAPK signaling pathway | 0.27 |
| **TAF15** | *FLJ46082* | NM_207417.1 | unknown | 0.07 |
|  | *ETV5* | NM_004454.1 | transcription factor | 0.11 |
|  | *EGR1* | NM_001964.2 | transcriptional regulator | 0.23 |
|  | *MAFB* | NM_005461.3 | regulation of hematopoiesis | 0.27 |
|  | *N.A.* | BM702416 | unknown | 0.29 |
|  | *FOS* | NM_005252.2 | cell proliferation, differentiation | 0.29 |
|  | *BRCA1* | NM_007295.2 | transcription, DNA repair | 0.30 |
|  | *CTGF* | NM_001901.1 | cell adhesion | 0.30 |
|  | *BMP2* | NM_001200.2 | growth factor; bone formation | 0.30 |
|  | *SPRY4* | NM_030964.2 | inhibitor of MAPK signaling pathway | 0.30 |
| **FUS+EWS+TAF15** | *ETV5* | NM_004454.1 | transcription factor | 0.15 |
|  | *SPRY4* | NM_030964.2 | inhibitor of MAPK signaling pathway | 0.15 |
|  | *SHISA2* | NM_001007538.1 | maturation of presomitic mesoderm cells | 0.18 |
|  | *ETV4* | NM_001986.1 | transcriptional activator | 0.20 |
|  | *BCKDHB* | NM_000056.2 | catabolism of branched-chain amino acids | 0.22 |
|  | *ETV4* | NM_001986.2 | transcriptional activator | 0.22 |
|  | *TCTN1* | NM_001082538.1 | modulate hedgehog signalling | 0.25 |
|  | *HIST1H2AC* | NM_003512.3 | member of the histone H2A family | 0.25 |
|  | *PCBD1* | NM_001001939.1 | phenylalanine hydroxylation | 0.26 |
|  | *C4orf34* | NM_174921.1 | unknown | 0.28 |
