# Supplementary material for: Gene Expression Responses to FUS, EWS, and TAF15 Reduction and Stress Granule Sequestration Analyses Identifies FET-Protein Non-Redundant Functions
Source: PLoS One. 2012 Sep 25;7(9):e46251. doi: 10.1371/journal.pone.0046251 (PMC3457980; doi:10.1371/journal.pone.0046251)
Supplement: Table S7 — IPA Bio Functions. Pathway analysis by IPA of DEGs in HEK293-cells after siRNA mediated gene knock-down of the FUS, EWS, TAF15, or FUS+EWS+TAF15 proteins. Five categories of the Bio Functions analysis belonging to each siRNA group are listed. (DOCX) [file pone.0046251.s014.docx]

| Supplementary Table S5. Top 5 identified IPA Bio Functions pathways. | | |  |
| --- | --- | --- | --- |
|  | | |  |
| siFUS | | |  |
| IPA category | **p-value** | **molecule** | |
|  |  |  |  |
| Cellular Development | 3,76E-07-2,43E-02 | HOXB2, OSTN, IL15RA, MPRIP, AGER, HES5, TBX6, AXIN1, PIK3R1, S100A4, SPRED2, ANK1, SPRY4, LUM, CAV1, NKX6-2, MCL1, HOXA2, NEUROD6, LCP1, PTP4A3, EN2, JUNB, MAFF, null, ARHGAP5, IRF7, AMH, ETV4, GNL3, ALAS2, CYR61, UBE2I, AKAP12, GEM, GADD45B, BMP2, RAB23, MCC, CDH23, SMPD1, APP, TNFRSF12A, SMURF1, STAP2, RPS6KA2, TERF1, NCKAP1, N4BP2L2, TXNIP, EGR1, EPHA3, GGT1, FUS, AFF1, FOS, BMF, CYP27B1, ADAM10, PDE5A, REC8, UBTF, PRDM16, ID4 |  |
|  |  |  |  |
| Cell Cycle | 3,28E-05-2,43E-02 | AKAP12, GADD45B, PIK3R1, BMP2, S100A4, APP, PLAC1, USP16, CAV1, RPS6KA2, TERF1, TXNIP, EGR1, CCNB3, JUNB, CYP1B1, FOS, IRF7, AMH, CAMKK1, REC8, GNL3, CYR61, AKAP9, ID4 |  |
|  |  |  |  |
| Cellular Growth and Proliferation | 1,54E-04-2,43E-02 | AKAP12, IL15RA, GEM, GADD45B, BACH2, AGER, AXIN1, NDRG2, PIK3R1, BMP2, S100A4, CDH23, PHC3, CD63, APP, TNFRSF12A, SPRY4, PLAC1, LUM, CAV1, STAP2, TERF1, MCL1, LCP1, TXNIP, PTP4A3, EGR1, JUNB, GGT1, MAFF, SULT2A1, CYP1B1, null, AFF1, FOSB, FOS, AMH, BMF, CYP27B1, ADAM10, PRR5, PDE5A, GNL3, KLC2, PRDM16, UBTF, CYR61, ID4 |  |
|  |  |  |  |
| Nervous System Development and Function | 1,54E-04-1,7E-02 | HOXB2, GEM, SLC6A9, HES5, AGER, MAP6, AXIN1, BMP2, S100A4, CDH23, SMPD1, APP, TNFRSF12A, NKX6-2, NCKAP1, HOXA2, NEUROD6, GAL3ST1, EGR1, JUNB, EN2, null, ARHGAP5, FOSB, FOS, AMH, ETV4, KCNJ5, ID4 |  |
|  |  |  |  |
| Cell Death | 2,4E-04-2,3E-02 | AKAP12, IL15RA, MPRIP, BACH2, GADD45B, AGER, AXIN1, BMP2, PIK3R1, S100A4, DDN, C1QA, SMPD1, APP, TNFRSF12A, PDE7B, ALDH1A3, CYP2E1, NKX6-2, CAV1, RPS6KA2, TERF1, NCKAP1, MCL1, TXNIP, EGR1, Prune2 (mouse), EN2, JUNB, GGT1, F3, null, FUS, CYP1B1, FOSB, FOS, KHDC1, AMH, SERINC3, BMF, NUAK1, PDE5A, GNL3, GNG2, UBTF, CYR61, MAP3K3, HFE |  |
| siEWS |  |  |  |
| IPA category | **p-value** | **molecule** | |
|  |  |  |  |
| Cell Morphology | 8,41E-07-8,81E-03 | GEM, STMN2, TSPAN7, DDIT3, PIK3R1, ATP4A, APP, DFFA, BCL2, VEGFA, TGM2, CALCB, DOK4, CCL28, CAV1, ATF4, VCL, null, PRKDC, TIAM1, PAK4, PSPN, EWSR1, POU4F2, TNC, ATF3, GAL3ST1, YWHAE, EGR1, EFNA3, TRIB3, ANXA2, ERBB3, CEBPB, JUNB, BAX, FDXR, CRMP1, FOS, PSD, SNAI2, CXCL12, FECH, HSP90AA1, SYN1, NEDD9 |  |
|  |  |  |  |
| Neurological Disease | 1,88E-06-8,85E-03 | HLA-DOA, TBX6, PIK3R1, ATP4A, CREB5, SCAMP5, FLJ35282, VEGFA, ST8SIA4, NEFM, CAV1, LARP1B, GAL3ST1, CHGB, ANXA2, JUNB, CA8, KCNB1, DUSP4, GNG2, BRE, ACTL6B, AP3B2, CD2BP2, DDIT3, TSPAN7, ASNS, TBCEL, TGM2, GADD45A, GARNL3, XCL1, KIAA1274, VCL, KHSRP, ATP6V1G2, PRKDC, PSMB9, PSPN, TNC, NELL2, null, MYOM2, CHGA, EGR1, UCN3, CERK, NBEA, EPHA3, PTGES, FOS, CRMP1, PTGS2, SYN2, NEDD9, ID4, SLC7A11, NDRG2, PKD1L2, UBE2V2, SPRED2, MOCOS, SGPP2, PCTP, SLITRK1, TIAM1, DTNBP1, EWSR1, YWHAE, MYH14, SYP, STAC, null, PREP, FOSB, RBKS, ETV4, RAB3C, HSP90AA1, CLDN14, SYN1, METTL9, ETV5, AADAT, LITAF, SLC1A4, STMN2, SLC38A1, APP, BCL2, CUL3, ZNF667, EHMT1, GNG4, CPNE9, GRIN1, PVALB, RNLS, RFX4, ERBB3, BAX, OLFM1, FDXR, CPLX1, SEPP1, GABBR2, KCNQ2, CXCL12, RDH12, PRDM16, C5orf13, BSN |  |
|  |  |  |  |
| Cellular Growth and Proliferation | 2,71E-06-9,14E-03 | SLC7A11, NDRG2, PIK3R1, SPRY4, VEGFA, CALCB, PRMT1, BOP1/LOC727967, TNFSF9, CAV1, ATF4, AMBN, null, TIAM1, EWSR1, ATF3, MYH14, ANXA2, JUNB, null, SLC3A2, FOSB, ATPIF1, SNAI2, RGL2, GEM, UNC5A, DDIT3, CAMK2N2, APP, BCL2, KISS1R, TGM2, GADD45A, SMOX, STAP2, XCL1, MAPKAPK2, GNG4, PRKDC, PAK4, PTPRK, POU4F2, TNC, VEGFB, CHGA, EGR1, CBX2, PPP1R15A, IKBKE, ERBB3, CEBPB, BAX, IL20RB, FDXR, FOS, MAD2L1, PTGES, ABTB1, CXCL12, PTGS2, PRDM16, C5orf13, NEDD9, ID4 |  |
|  |  |  |  |
| Cell Death | 6,15E-06-8,12E-03 | SLC7A11, PIK3R1, DFFA, VEGFA, MAP3K10, CALCB, PPP1R3F, ST8SIA4, TNFSF9, PPFIA4, CAV1, ATF4, SNN, null, TIAM1, EWSR1, ATF3, YWHAE, Prune2 (mouse), JUNB, null, FOSB, PPP1R13L, SNAI2, HSP90AA1, DUSP4, GNG2, BRE, UNC5A, DDIT3, TNFAIP3, CAMK2N2, ASNS, APP, BCL2, CUL3, TGM2, GADD45A, ALDH1A3, XCL1, SMOX, VCL, ATP6V1G2, PRKDC, GRIN1, PAK4, POU4F2, PSPN, PTPRK, TNC, EGR1, EFNA3, PPP1R15A, IKBKE, TRIB3, ERBB3, CERK, CEBPB, BAX, FDXR, PTGES, FOS, MAD2L1, CXCL12, CYB5A, PTGS2, NEDD9, ID4 |  |
|  |  |  |  |
| Cell Cycle | 1,05E-05-9,14E-03 | DDIT3, PIK3R1, MYLK2, CAMK2N2, ASNS, APP, BCL2, VEGFA, CUL3, CALCB, PRMT1, BOP1/LOC727967, GADD45A, USP16, CAV1, KIF25, PRKDC, ATF3, EWSR1, PTPRK, TNC, YWHAE, MYH14, EGR1, CBX2, PPP1R15A, RAD51L1, ERBB3, CEBPB, BAX, JUNB, FOS, MAD2L1, ABTB1, CXCL12, HSP90AA1, RGL2, PTGS2, NEDD9, BRE, ID4 |  |
| siTAF15 |  |  |  |
| IPA category | **p-value** | **molecule** |  |
|  |  |  |  |
| Cellular Development | 4,2E-08-7,38E-03 | OSTN, SLC7A11, CTGF, ITGB1BP1, PIK3R1, ITGA8, RBP1, ANK1, SPRY4, VEGFA, EN1, RARA, IFITM2, HOXA10, EBF2, BRCA1, TMBIM1, WT1, FGFR1, RAC1, MEOX1, MBD2, CITED1, MAFF, SRSF3, ARHGAP5, ZNF423, MAX, ETV4, RASSF4, PML, SOCS5, AKAP12, GEM, BMP2, BCL11B, MAFG, SHROOM2, HMOX1, ID1, null, GNAT2, AKT3, GATA6, TERF1, PLAT, AGFG1, TNC, EGR1, NQO1, MAPK9, TERC, CALCA, FOS, GPER, CXCL12, PAG1, MAFB, PTGS2, PRKCB, FNDC3A |  |
|  |  |  |  |
| Cellular Movement | 2,09E-07-7,18E-03 | SULF1, GEM, CTGF, SCN2B, BMP2, ITGB1BP1, PIK3R1, UNC5B, CCBP2, SPRY4, VEGFA, ID1, HMOX1, LGMN, L3MBTL1, RARA, GATA6, AKT3, EBF2, NEDD4L, PIK3IP1, PLAT, GRIN1, TNC, TPST2, RHOC, EGR1, BGN, FGFR1, NQO1, RAC1, MAPK9, MBD2, F3, TERC, CALCA, ARHGAP5, FOS, SCGB1A1, MAX, CXCL12, ETV4, MAFB, PTGS2, NEDD9, ETV5, PRKCB |  |
|  |  |  |  |
| Cell Cycle | 7,25E-07-7,18E-03 | AKAP12, SULF1, PIK3R1, BMP2, MYLK2, VEGFA, GPR3, HMOX1, ID1, L3MBTL1, RARA, HOXA10, GATA6, BRCA1, NEDD4L, TERF1, WT1, TP53INP1, TNC, EGR1, FGFR1, RAC1, MAPK9, TERC, FOS, MAX, CXCL12, PTGS2, PML, NEDD9, SOCS5, PRKCB |  |
|  |  |  |  |
| Cell Death | 2,27E-05-7,38E-03 | SLC7A11, CTGF, PIK3R1, UNC5B, RBP1, EN1, VEGFA, PPP1R3F, CYP2E1, RARA, SNN, BRCA1, WT1, HBXIP, TP53INP1, FGFR1, DIDO1, RAC1, TUB, FOSB, ZNF423, MAX, KHDC1, RASSF4, PML, AKAP12, SULF1, BMP2, BCL11B, null, HMOX1, ID1, AKT3, GATA6, TERF1, PIK3IP1, PLAT, GRIN1, TNC, LUC7L3, RHOC, BGN, EGR1, NQO1, EPX, MAPK9, DNAJB9, TERC, F3, UACA, CALCA, FOS, CXCL12, PTGS2, MAFB, NEDD9, PRKCB |  |
|  |  |  |  |
| Genetic Disorder | 6,33E-05-1,95E-03 | HPCAL4, CTGF, IDS, MAP6, ABAT, PIK3R1, MYLK2, ITGA8, BEX5, ADAMTS2, VEGFA, GNB4, LGMN, APLP1, RARA, IFITM2, EBF2, PKIA, KCNJ12, CACNA1G, WT1, TP53INP1, CRELD1, MBD2, USP6, MAFF, TMEM192, ZNF423, CERKL, AKAP12, null, BCL11B, IDH1, FAM183A, ID1, KIAA1274, PLAT, PLOD2, TNC, RHOC, EGR1, DIP2A, PRELID2, F3, FOS, SCGB1A1, FA2H, RBMS3, SLC22A18, MAFB, PTGS2, NEDD9, LONRF2, COLQ, PRKCB, FNDC3A, SCN2B, SLC7A11, CSNK1G1, UNC5B, INA, RBP1, ANK1, C10orf72, CYP2E1, HOXA10, NEDD4L, BRCA1, SLCO4C1, GAS5, FGFR1, RAC1, SCOC, TUB, UBXN11, SRSF3, ETV4, PML, SOCS5, ETV5, SULF1, BMP2, SHROOM2, HMOX1, null, CCDC27, GNAT2, AKT3, GATA6, TERF1, PIK3IP1, GRIN1, LRRN2, TPST2, C9orf5, TAF15, BGN, EPX, SMOC1, NQO1, MAPK9, DNAJB9, TERC, CPLX1, APOLD1, CALCA, BEST1, PPAPDC1A, GPR56, POLR3B, GPER, ACADVL, RAB36, CXCL12, C8orf34, DOCK10 |  |
| siFUS+siEWS+siTAF15 | | |  |
| IPA category | **p-value** | **molecule** |  |
|  |  |  |  |
| Cell Morphology | 1,69E-04-2,43E-02 | IL8, MPRIP, CAPZB, GFRA3, EGR1, ACTA2, ABL1, NFKB2, FOXG1, CCND1, FOS, ID1, SEMA4D, TLR5, GAB1, LAMA4, CAV1, DTNA, CD82, PRKCB |  |
|  |  |  |  |
| Cellular Development | 1,69E-04-2,43E-02 | GRIN2A, SLC7A11, CTGF, MPRIP, BAD, NFATC3, ITGB1BP1, DIRAS3, LIMK2, MLL2, ZNF536, SPRED2, RBP1, CCND1, TBX18, SPRY4, PRMT1, RORA, OLIG2, RARA, HDAC7, CAV1, LHX3, WNT5B, TNFSF13B, PDK4, HOXA2, IL8, WT1, ATG7, EWSR1, FOXP1, PCDH15, MEOX1, NFKB2, EN2, FOXG1, MAFF, null, MECOM, SRSF3, ZNF423, GAB1, AMH, ETV4, EPCAM, HSP90AA1, ALAS2, REL, ENC1, RORB, HIST1H1C, OSGIN1, GFRA3, BMP2, TBXA2R, CDH23, ABL1, HDAC9, CTLA4, COMP, ID1, TNFRSF6B, NCKAP1, PLAT, MAPKAP1, CAPZB, null, MTCP1, EGR1, CD36, HYAL1, CBX2, DEDD, FUS, FOS, SEMA4D, TLR5, LAMA4, BMF, ZEB2, CD82, GCM1, null, TBX1, ID4, FOXC2, PRKCB |  |
|  |  |  |  |
| Cellular Growth and Proliferation | 4,27E-04-2,43E-02 | SLC7A11, CTGF, BAD, NFATC3, DIRAS3, MLL2, ABCG1, CCND1, TBX18, PLCD3, PRMT1, OLIG2, RARA, HDAC7, CAV1, CD84, ARHGAP24, null, WNT5B, TNFSF13B, WT1, IL8, TP53INP1, EWSR1, FOXP1, NFKB2, FOXG1, null, MECOM, AMH, CYCS, EPCAM, REL, FOXK1, ANG, OSGIN1, GFRA3, BMP2, TBXA2R, ABL1, FKBP1A, MSLN, CTLA4, ID1, NFAT5, TNFSF15, TNFRSF6B, PLAT, PTPRK, EGR1, CD36, CBX2, IL20RB, FUS, FOS, SEMA4D, TLR5, LAMA4, CD82, null, ID4, PRKCB |  |
|  |  |  |  |
| Neurological Disease | 5,89E-04-2,43E-02 | SLC25A26, GTPBP1, CTGF, BEX5, CREB5, ZNF385D, SCN4A, OLIG2, CYTH1, RARA, SYPL1, CAV1, PRPF40A, ZBTB20, TCERG1, LARP1B, KLF12, C20orf196, WT1, APTX, STRC, ATG7, FOXP1, KIAA1712, PCDH15, NFKB2, FOXG1, MAFF, HSD17B6, AGPAT4, null, ZNF423, GAB1, ABCC5, UQCRC2, GLRA1, DTNA, CYCS, ALAS2, ENC1, ANG, FOXK1, FCGRT, FKBP1A, MAP4, SNRPD3, TNFRSF6B, B4GALT5, PLAT, MBD5, DCDC5, PLOD2, SPEG, null, PSPN, MYOM2, EGR1, CERK, TMLHE, FOS, PDZD2, PGCP, SEMA4D, FGGY, ZEB2, TAGLN, null, GLS2, ID4, PRKCB, GRIN2A, SLC7A11, ARHGAP26, CLN8, BAD, EIF6, VPS13A, MLL2, PIP5K1B, SPRED2, PLCD3, RORA, KLHL3, TTLL11, IL8, EWSR1, SCOC, null, MECOM, FGF17, ETV4, OR2A5, TRPS1, HSP90AA1, REL, ETV5, OBFC1, SLC35D3, ACTA2, ABL1, RPL22L1, CDH23, ERC1, SLC38A1, CTLA4, OPRL1, BCYRN1, null, SNCB, METAP1D, GPR83, PVALB, CAPZB, HIPK3, ATP2C1, ATRX, CD36, KCNMB3, OLFM1, NAP1L5, PIGG, AK5, KCNQ2, TLR5, GPR137B, PPM1B, ELOVL2, IKBKAP, GCM1, ZNF365 |  |
|  |  |  |  |
| Cell Death | 7,89E-04-2,43E-02 | GRIN2A, CTGF, BAD, ABCG1, RBP1, CCND1, MTMR8, PLCD3, PPP1R3F, CYP2E1, RARA, PPFIA4, CAV1, null, TNFSF13B, IL8, WT1, ATG7, EWSR1, FOXP1, EN2, NFKB2, RAD51L3, null, MECOM, ZNF423, AMH, GAB1, TRPS1, CYCS, HSP90AA1, REL, HIST1H1C, OSGIN1, ERN1, GFRA3, BMP2, TBXA2R, ABL1, HDAC9, FKBP1A, CTLA4, COMP, ID1, MAP4, CLDN4, FIGLA, SNCB, TNFRSF6B, SERBP1, PLAT, PTPRK, null, ATG10, ATP2C1, EGR1, ATRX, CD36, CERK, MTMR12, FOS, AK5, PPM1B, LAMA4, BMF, CD82, ID4, PRKCB |  |
|  |  |  |  |
